# Diclofenac Detection via Inner‐Filter Effect Using Pyridine‐Modified Triphenylene

**DOI:** 10.1002/cplu.202400687

**Published:** 2025-06-29

**Authors:** Navdeep Kajal, Amar Raj, Rajendhraprasad Tatikonda, Jan Lundell, Matti Haukka

**Affiliations:** ^1^ Department of Chemistry University of Jyväskylä P. O. Box 35 Jyväskylä FI‐40014 Finland; ^2^ Nanoscience Center Department of Chemistry University of Jyväskylä P. O. Box 35 Jyväskylä FI‐40014 Finland

**Keywords:** diclofenac, fluorescence quenching, hexa‐pyridine triphenylene, inner‐filter effects, sensors

## Abstract

Triphenylene derivatives **3PY** (2,3,6,7,10,11‐hexa(pyridine‐3‐yl)triphenylene) and **4PY** (2,3,6,7,10,11‐hexa(pyridine‐4‐yl)triphenylene) are synthesized using a Suzuki–Miyaura coupling protocol and evaluated as potent chemosensors for the detection of diclofenac in solution phase. Screening against various commonly used nonsteroidal antiinflammatory drugs and hormones demonstrates that the derivatives are selective for diclofenac. The fluorescence quenching mechanism is attributed to the inner filter effect because of the overlap between the UV–vis absorption spectrum of diclofenac and the excitation spectra of triphenylene derivatives. They can sense diclofenac with a low limit of detection of 1.18 μM, offering a simple and cost‐effective method. Moreover, the practicability and applicability of the proposed sensor are confirmed in the lake water with reliable recovery and precision.

## Graphical abstract

The synthesis of hexa‐pyridine triphenylene derivatives via Suzuki‐coupling reaction and their application toward the sensing of diclofenac have been studied for the first time. The **3PY** and **4PY** are selective for diclofenac over the other commonly prescribed pharmaceuticals and hormones tested.

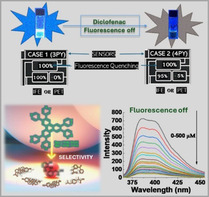

## 1 Introduction

**Figure 1:**
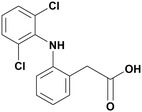
Molecular structure of the DCF drug.

Diclofenac (DCF), example 2‐(2‐((2,6‐dichlorophenyl) amino) phenyl) acetic acid, is a commonly prescribed nonsteroidal antiinflammatory drug (NSAID). It has antipyretic and analgesic properties and is broadly used in human and veterinary medicines.^[^ ^1^ ^]^ Musculoskeletal problems, such as rheumatoid arthritis, polymyositis, osteoarthritis, dermatomyositis, tooth pain, and ankylosing spondylitis, are among the disorders.^[^ ^2^ ^]^ It is immediately absorbed by the organism and has a half‐life of just 1–2 h.^[^ ^3^ ^]^ The primary mechanism underlying its analgesic, antithermal, and antiinflammatory effects is the suppression of cyclooxygenase enzyme, which prevents prostaglandin from being synthesized.^[^ ^3^ ^]^ Due to its increased solubility, DCF's sodium salt is typically used, and the hepatic enzyme readily metabolizes the DCF. It has two benzene rings in its structure (**Figure** **1**), which can interact with other aromatic systems through π–π interaction. In contrast, the secondary amine and carboxylic functional group of DCF can participate in hydrogen bond formation.

The occurrence and sustained presence of hormones and pharmaceutical compounds in water bodies has raised concerns about their negative effects on both the marine environment and human health.^[^ ^4^, ^5^ ^]^ For instance, due to inadequate removal during water treatment, DCF is found in extremely high amounts in the effluent of water treatment plants.^[^ ^6^ ^]^ Moreover, various research groups have reported the presence of DCF in several environmental samples, such as drinking water, surface water, and wastewater.^[^ ^7^, ^8^, ^9^, ^10^, ^11^ ^]^ Due to its adverse effects and the extensive commercial use in medical, pharmaceutical, and biomedical applications, it is important to develop a feasible method to detect DFC at trace levels in different matrices. To date, various analytical methods have been suggested and applied for the detection of DCF, including gas chromatography–mass spectrometry,^[^ ^12^ ^]^ potentiometric method,^[^ ^13^ ^]^ thin‐layer chromatography,^[^ ^14^ ^]^ liquid chromatography,^[^ ^15^ ^]^ and voltammetry methods.^[^ ^16^ ^]^ All the previously mentioned techniques have their strengths but also some shortcomings; they are often laborious, slow, and require complex, sophisticated instruments. In contrast, fluorescence probes have gained popularity due to their accuracy, ease of use, affordability, and fast detection times.^[^ ^17^, ^18^, ^19^, ^20^ ^]^ Moreover, the mechanism of sensing found in fluorescent sensors was Förster resonance energy transfer (FRET), photoinduced electron transfer (PET), static or dynamic quenching or ground‐state charge transfer complexation, indicator displacement assay based or a combination of two or more mechanisms.^[^ ^19^, ^21^, ^22^ ^]^ In all the above‐reported mechanisms, there is some kind of interaction between the probe and the analytes. As compared to the other mechanisms, inner filter effect (IFE) based sensing works in a straightforward manner, devoid of any kind of interaction between the fluorophore (probe) and the absorber (analyte).^[^ ^23^ ^]^ The IFE occurs when the absorption spectrum of the absorber (DCF) in the detection system overlaps the excitation and/or emission spectrum of the fluorophore (**3PY** and **4PY**). IFE is a crucial nonirradiation energy conversion model in spectrofluorometry, which arises from the absorption of the excitation and/or emission light by the absorber in the detection system.^[^ ^24^ ^]^ To design an IFE‐based sensing system, there should be an analyte having sensitive absorption and a fluorophore having analyte‐independent fluorescence, and the excitation and emission spectra of the fluorophore could be modulated by the absorption spectrum of the absorber (quencher or analyte).^[^ ^24^ ^]^ Therefore, IFE‐based sensing platforms offer simple and flexible sensing systems without any restricted chemical bond between the fluorophore and quencher.

Triphenylene‐based materials have gained a lot of attention due to their optoelectronic properties and capability to undergo both bulk and solution phases.^[^ ^25^ ^]^ The ability of triphenylene derivatives to self‐assemble in the solution phase is a crucial fabrication technique for creating complex functional structures.^[^ ^26^ ^]^ However, because of effective intermolecular π–π stacking, these triphenylene derivatives produce nonfluorescent H‐aggregates in solution.^[^ ^27^, ^28^ ^]^ In contrast, luminous π‐conjugated organic molecules are needed for the fabrication of optical and electronic devices such as organic light‐emitting diodes (OLEDs), OLE field‐effect transistors, and organic fluorescent sensors.^[^ ^29^, ^30^ ^]^

**Figure 2:**
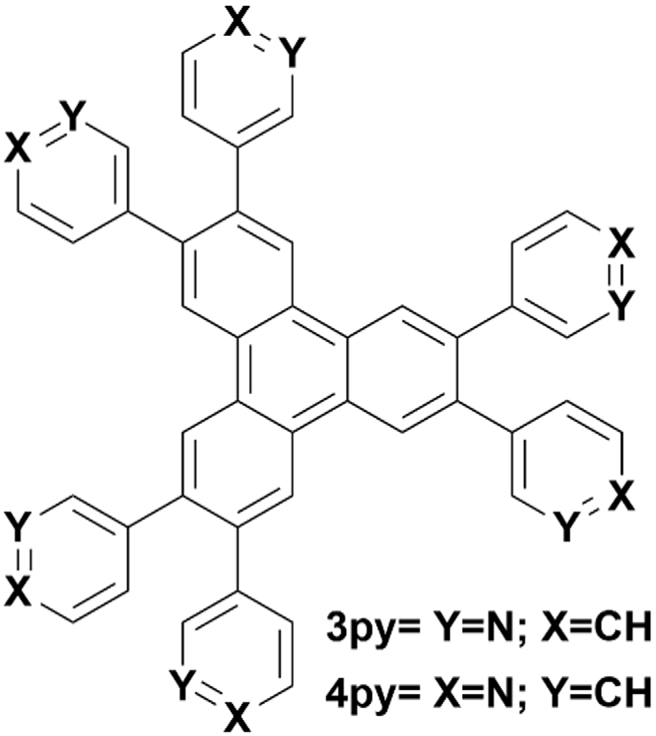
Chemical structure of pyridine‐modified triphenylene derivatives.

In this work, novel pyridine‐modified triphenylene derivatives were designed and synthesized with nitrogen located at *meta* (**3PY**) or *para* (**4PY**) positions, respectively (**Figure** **2**). Our goal was to develop a selective method for sensing DCF based on quenching of the fluorescence intensity of pyridine‐modified triphenylene compounds. The fluorescence intensity of derivatives (387 nm) is quenched by the IFE process due to the overlapped absorption spectra of DCF with the excitation spectra of triphenylene derivatives. Moreover, a series of commonly prescribed NSAIDs and hormones were also tested to show the selectivity of the sensor, such as naproxen, progesterone, estrone, ibuprofen, testosterone, acetylsalicylic acid (aspirin), paracetamol, ethinyl estradiol, ketoprofen, and β‐estradiol. To our best knowledge, this is the first report of triphenylene or any of its derivatives being employed as a selective chemosensor for pharmaceuticals. This fluorescence method has been further validated by testing lake water spiked with DCF, indicating its high potential for facile and sensitive detection of DCF.

## 2 Experimental Section

### 2.1 Materials

All solvents and reagents were purchased from Sigma‐Aldrich and TCI Europe and used as received without further purification. Triphenylene (Sigma‐Aldrich, 98%), iron reduced powder (Sigma‐Aldrich, >99%), liquid bromine (Sigma‐Aldrich, ≥99.5%), DCF (TCI, >98%), naproxen (TCI, >98%), progesterone (TCI, >98%), estrone (TCI, >98%), ibuprofen (TCI, >98%), testosterone (TCI, >98%), acetylsalicylic acid (TCI, >98%), paracetamol (TCI, >98%), ethinyl estradiol (TCI, >98%), ketoprofen (TCI, >98%), β‐estradiol (Sigma‐Aldrich, ≥98%).

### 2.2 Synthesis of Hexa‐Bromotriphenylene

A solution of 4.95 mmol triphenylene (1.13 g) in nitrobenzene (40 mL) was prepared in a round‐bottomed flask. Catalytic iron shavings (1.78 mmol, 100 mg) were added, and the reaction flask was equipped with a dropping funnel. Liquid bromine (42.6 mmol, 2.2 mL) was added dropwise slowly over 10 min. The resulting solution was left to stand for 24 h, then refluxed at 200 °C for 2 h. The reaction solution was allowed to cool to room temperature, and then, diethyl ether was added (150 mL), and the resulting solid precipitate was filtered and rinsed with diethyl ether (2 × 30 mL). The white solid was filtered, then dried in an oven at 80 °C. The resulting off‐white solid product was used without further purification.

### 2.3 Synthesis of 3PY and 4PY

**Figure 3:**
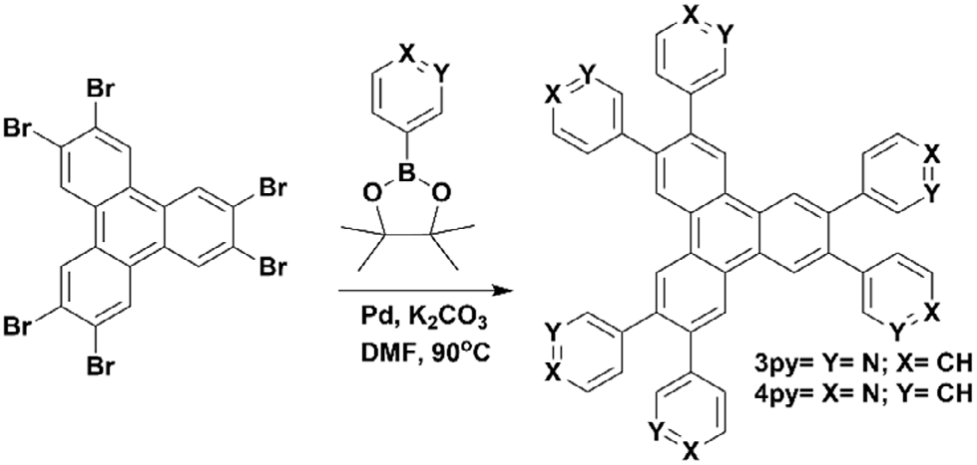
Schematic diagram for the synthesis of **3PY** and **4PY**.

Pyridine‐modified triphenylene derivatives (**3PY** and **4PY**) were synthesized by following Suzuki‐Miyaura coupling reactions (**Figure** **3**). To the mixture of suspension of 3‐boronic acid pinacol ester (1.231 g, 6 mmol), Tetrakis triphenylphosphine)‐palladium (0) (0.115 g, 0.1 mmol) in DMF (20 mL) was added a suspension of 2,3,6,7,10,11‐hexabromotriphenylene (0.701 g, 1 mmol) in an aqueous solution (2 mL) of potassium carbonate (1.656 g, 12 mmol). The mixture was refluxed overnight at 90 °C under N_2_ and allowed to cool to room temperature. The product was in aqueous phase and extracted with CHCl_3_. Further, the product was recrystallized in dimethyl sulfoxide (DMSO) to make it pure and, later, used for sensing applications. Yield = 79%. ^1^H NMR (300 MHz, D_2_O + DCl, δ ppm) 9.124 (s, 6H), 9.006 (s, 6H), 8.774 (d, 6H), 8.555 (d, 6H), 8.012 (t, 6H). ^13^C NMR (75 MHz, D_2_O + DCl + CD_3_OD), δ ppm) 149.273, 142.638, 141.564, 139.644, 134.192, 131.538, 128.688, 128.358 (Figure S3 and S4, Supporting information). The structure of compound **3PY** was further confirmed by a matrix assisted laser desorption/ionization time‐of‐flight (MALDI‐TOF) mass spectrum, which showed a parent ion peak at m/z 691.26 (M + H)^+^ (Figure S1, Supporting Information). Moreover, C—N and C=N bonds in the compound were further confirmed by Fourier transform infrared spectroscopy (FTIR). (Figure S7, Supporting Information).

In the case of **4PY**, 4‐boronic acid pinacol ester (1.231 g, 6 mmol) was used as a reagent, and the product was in the black precipitate. Further, the product was recrystallized in DMF to make it a pure white powder and, later, used it for sensing applications. Yield = 44%. ^1^H NMR (300 MHz, D_2_O + DCl, δ ppm) 9.248 (s, 6 H), 8.810 (d, 12 H), 8.191 (d, 12 H), ^13^C NMR (75 MHz, D_2_O + DCl + CD_3_OD), δ ppm), 158.278, 142.202, 136.132, 131.937, 129.478, 128.885 (Figure S5 and S6, Supporting Information). MALDI‐TOF MS m/z: 346.13 (M+2 H)^2+^(Figure S2, Supporting Information). Moreover, C—N and C=N bonds in the compound were further confirmed by FTIR. (Figure S8, Supporting Information).

### 2.4 Preparation of Sample Solution for Analysis

DMSO was used for the preparation of a stock solution of **3PY** and **4PY** (50 μM), which was further diluted to 5 μM concentration for spectral analysis. Stock solutions (5.0 × 10^−3^ M) of various drugs and hormones, including DCF, naproxen, progesterone, estrone, ibuprofen, testosterone, acetylsalicylic acid, paracetamol, ethinyl estradiol, ketoprofen, and β‐estradiol, were prepared in ethanol. The solutions were prepared just before taking absorbance and emission measurements. All measurements were recorded at room temperature.

### 2.5 Spectroscopic Instrumentation

The UV–vis absorption studies were carried out on a Perkin–Elmer Lambda 650 UV–vis spectrometer using a quartz cuvette with 10 mm optical path length. Fluorescence emission and excitation measurements were carried out on an Agilent Cary Eclipse fluorescence spectrometer with a similar cuvette. Fluorescent decay profiles were measured with the time‐correlated single photon counting (TCSPC) setup based on the HydraHarp 400 (PicoQuant) TCSPC module. The excitation source consisted of a PicoQuant PDL 800‐D pulsed diode laser driver and a diode laser head (LDH‐PC‐375) emitting light at 375 nm wavelength. More details about TCSPC are in Section 2 of ESI. Electrospray ionization mass spectrometry (ESI‐MS) experiments were performed on a 653 °C Q‐TOF mass spectrometer. ^1^H and ^13^C NMR were performed by Bruker NMR 300 with a broad band fluorine observe (BBFO) probe using D_2_O and DCl as solvents and CD_3_OD as an external reference for ^13^C NMR. Data were reported as follows: chemical shift in ppm (δ). The chemical environment of the derivatives was studied by FTIR‐attenuated total reflectance (ATR) Bruker Alpha equipped with diamond crystal in the range 400–4000 cm^−1^.

### 2.6 Electrochemical Measurements

The cyclic voltammetry (CV) apparatus used was a computer‐controlled IVIUMSTAT instrument. The hardware used consisted of a potentiostat with a rate‐determined scan capability. The software allowed us to display a current (Amperes, A) versus potential (voltage, V) response for the analyte solution. A three‐electrode system was used to generate the current potential profile. These electrodes consist of a working electrode (7 mm diameter) of glassy carbon, a platinum wire as a counter electrode, and an Ag/AgCl reference electrode. The Ag/AgCl reference electrode was initially calibrated using the ferrocenium/ferrocene pseudoreference. A 50 mL of 0.1 M Bu_4_NPF_6_ in acetonitrile solution was used as an electrolyte, and the solution of **3PY**/**4PY**/DCF was prepared beforehand. Zetapotential measurements were carried out with the Malvern Zetasizer Ultra ZS90 instrument (Malvern Instruments Ltd., UK). The zeta potential reported here is the average of 12 scans.

## 3 Results and Discussion

### 3.1 Optical Properties

**Figure 4:**
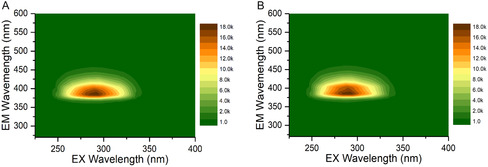
A) 3D plot for **3PY** sample (5 μM) where excitation and emission varied. B) 3D plot for **4PY** sample (5 μM). The instrument parameter for both is the same. Excitation varied from 225–400 nm, and the emission range varied from 270–600 nm. The legend and color palette denote the intensity of the peak. The blank was subtracted with the DMSO solvent.

**Figure 5:**
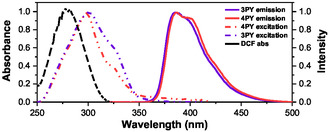
Absorbance, excitation, and emission spectra are plotted for DCF and **3PY**/**4PY** sensors. The fluorescence spectra of **3PY**/**4PY** (5 μM) were collected at the excitation wavelength of 300 nm in DMSO. All the spectra here are normalized by dividing by the maxima.

**Figure 6:**
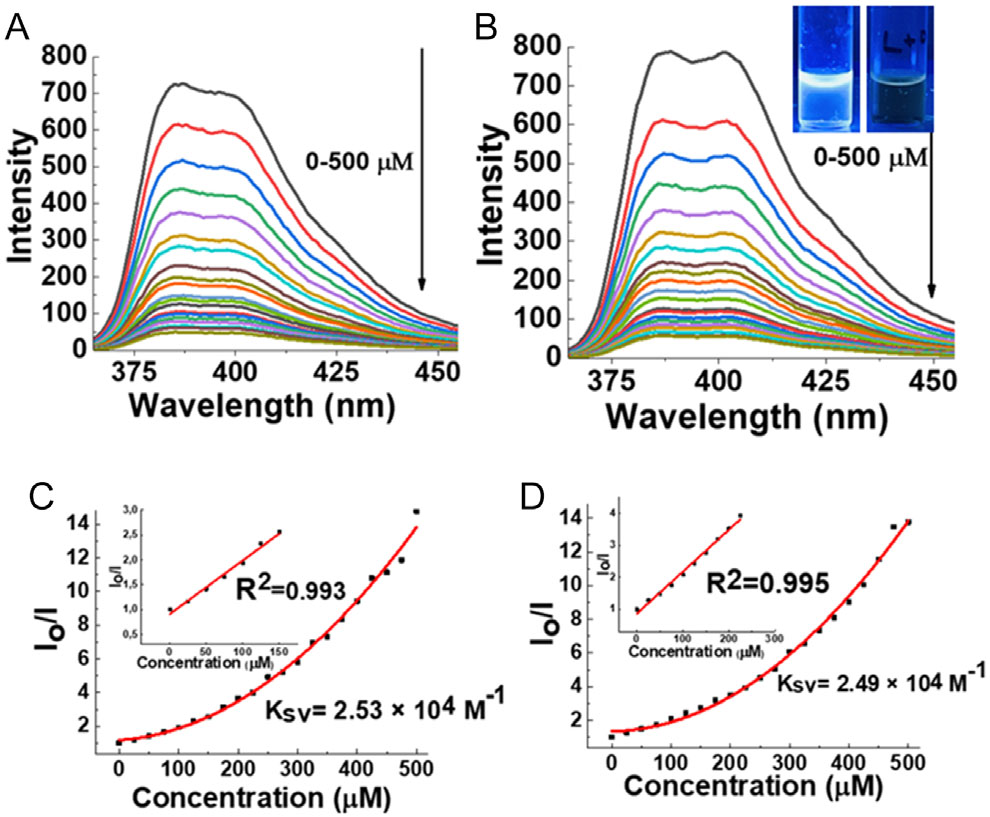
A) Emission spectra of **3PY** (5 μM) and B) **4PY** (5 μM) in DMSO upon addition of DCF (0–500 μM). C) Stern–Volmer plot for **3PY** and D) **4PY,** and the inset shows a linear relationship between fluorescence intensity and concentration of DCF. (*λ*_ex_ = 300 nm).

The optical properties of the triphenylene derivatives were investigated using UV–vis absorption and fluorescence spectroscopy. The UV–vis absorption spectrum (Figure S12, Supporting information) shows a prominent absorption peak centered at 294 nm, which can be attributed to the π–π* transition of the C=C bonds in the aromatic *sp* ^2^ domains. The derivatives, **3PY** and **4PY**, exhibited high quantum yields of ≈22.3% and 25.6%, respectively (Figure S12, Supporting Information), indicating strong fluorescence under UV light. The quantum yield was calculated with the reference of quinine sulfate, and the protocol is mentioned in Section 3 of ESI. The molar extinction coefficient for **3PY** and **4PY** was calculated at 132000 ± 2000 M^−1^ cm^*−*1^ and 120000 ± 2000 M^−1^ cm^*−*1^, respectively, as shown in Figure S11, Supporting Information. In daylight, the solutions of **3PY** and **4PY** were colorless and transparent, but under a 365 nm UV lamp irradiation, they emitted a bright blue light visible to the naked eye, as illustrated in Figure 6B (inset). The emission spectra of these derivatives were recorded under various excitation wavelengths (Figure S13C and S13D, Supporting Information), and it was observed that the wavelength of the emission maximum of both **3PY** and **4PY** remained independent of the excitation wavelength, although the intensity varied depending on the excitation wavelength, reaching a maximum at ≈300 nm (as shown in **Figure** **4**). Despite the changes in excitation wavelength, the emission wavelengths of **3PY** and **4PY** remained nearly constant, centered at ≈387 nm. The excitation spectra of **3PY** and **4PY**, shown in **Figure** **5**, are centered around 300 nm, with the **3PY** spectrum exhibiting a slight blue shift of about 1 nm relative to the **4PY** excitation spectrum, and the excitation spectrum of **3PY** has a higher full width at half maximum. This minor shift and broadening underscore the subtle differences in the optical behavior of the two derivatives.

### 3.2 Sensitive Detection of DCF

The fluorescence titrimetric method was used to measure the concentration‐dependent luminescence intensity, as seen in **Figure** **6**A,B, upon the addition of DCF (0–500 μM.), there is a quenching of fluorescence in the emission spectra of **3PY** and **4PY**, demonstrating the high sensitivity of the sensor. The luminescence intensity of **3PY** and **4PY** was completely quenched, and the fluorescence emission of the analytical system decreased proportionally to the increase in the DCF concentration. The amount of DCF was detected by calculating the fluorescence‐quenching efficiencies. The quenching of **3PY** and **4PY** was studied by Stern–Volmer plot (Figure 6C,D), and from there the value of the Stern–Volmer constant (K_SV_), generally known as the quenching constant, was determined. The Stern–Volmer plot was found to be linear at lower concentrations of **3PY** and **4PY** (Figure 6C,D inset) up to 150 and 225 μM with correlation coefficient (R^2^), 0.993 and 0.995, respectively. At higher concentrations, the divergence or nonlinear nature of the S–V plot indicates amplified quenching in **3PY** and **4PY**. This could be due to the presence of static and dynamic quenching mechanisms, efficient singlet exciton migration, self‐absorption, other additional energy transfer processes, or IFE caused by the higher concentration of the quencher.^[^ ^31^ ^]^

The fluorescence quenching could be described by the Stern−Volmer equation, I_0_/I = 1 + K_SV_ [M], where I_0_ is the initial fluorescence intensity of the triphenylene derivatives, I is the fluorescence intensity after the addition of DCF, K_sv_ is the Stern–Volmer constant, and M is the concentration of the DCF. The values of Stern–Volmer constants are 2.53 × 10^4^ M^−1^ and 2.48 × 10^4^ M^−1^ of **3PY** and **4PY**, respectively. The detection limit of the compound is determined based on the empirical formula given below

$$\text{Limit of detection }\left(\text{LOD}\right){\text{ = 3β/K}}_{\text{SV}}$$

where β = standard deviation (SD) of the blank sample and K_SV_ is the slope of the calibration curve. SD is calculated from the measurements of fluorescent spectroscopy. The normalized SD of the blank sample (5 measurements) is 0.00995 and 0.00992 for 3PY and 4PY, respectively (where the initial fluorescent value (F_0_) = 1). By using the above empirical formula, the LOD for DCF is 1.18 and 1.20 μM from **3PY** and **4PY,** respectively.

### 3.3 Selectivity Studies

For optimal optical performance, an excellent fluorescent probe should also have high selectivity toward the desired target. Therefore, different commonly prescribed pharmaceuticals and hormones were also tested to study the selectivity of the sensor. Only in the presence of DCF, the luminescence intensity of **3PY** and **4PY** was completely quenched, whereas, in the presence of other pharmaceutical drugs and hormones, the quenching effect was very minute, as shown in Figure S13E and S13F, Supporting Information.

**Figure 7:**
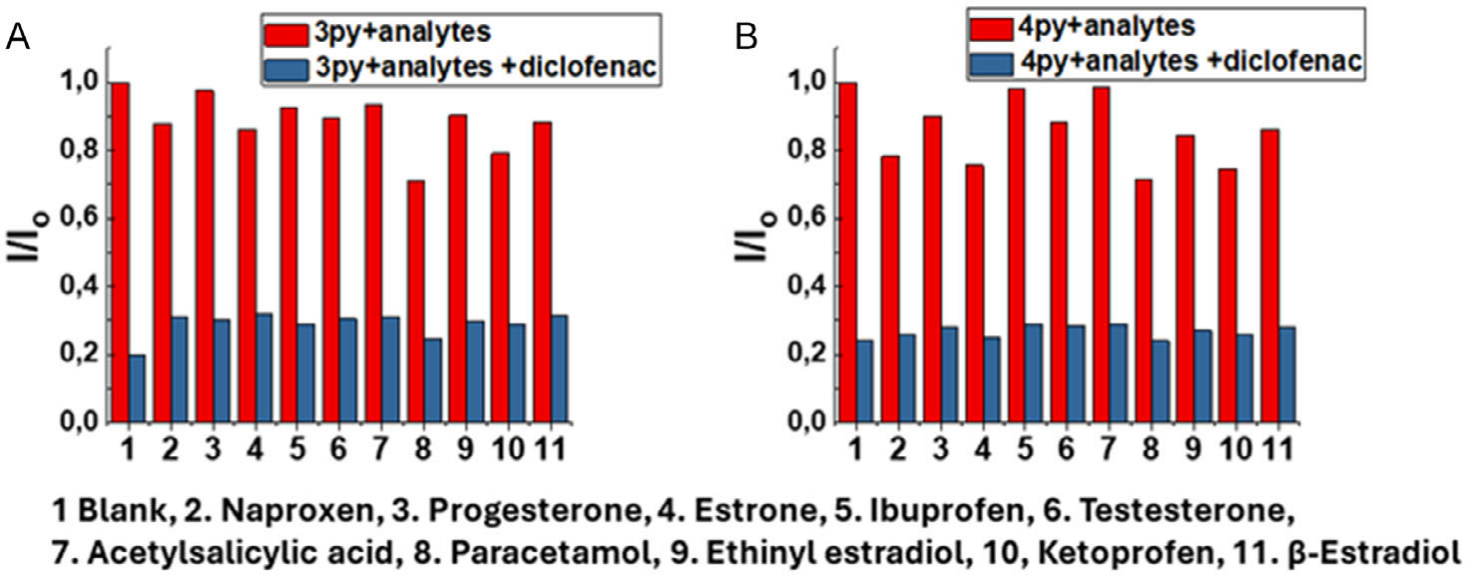
A) Relative changes in the fluorescent intensity of **3PY** (5 μM) and B) **4PY** (5 μM) in the presence of DCF (250 μM) and other pharmaceutical drugs and hormones (500 μM).

The selectivity of **3PY** and **4PY** was investigated by analyzing the fluorescence intensities in solutions that contained other pharmaceuticals and hormones. For this, different hormones and pharmaceutical compounds were added to the sample solutions. A typical sample contained additional NSAIDs and hormones such as naproxen, ibuprofen, acetylsalicylic acid (aspirin), paracetamol, ketoprofen, progesterone, estrone, β‐estradiol, ethinyl estradiol, and testosterone. Only DCF showed considerable fluorescence quenching, while other organic compounds showed significantly less fluorescence quenching. This indicates clearly that triphenylene derivatives used in this study could act as an efficient and selective fluorescence sensor for the sensing of DCF (**Figure** **7**).

### 3.4 Sensing Mechanism

The fluorescence quenching of **3PY** and **4PY** can occur through several pathways, including FRET, PET, excited‐state reactions, the IFE, static quenching (e.g., ground‐state donor–acceptor complexation), aggregation‐induced quenching, and dynamic quenching (e.g., collisional/diffusional quenching), as suggested by previously reported literature.^[^ ^24^, ^32^, ^33^, ^34^ ^]^ In this section, we analyze the steady‐state spectra and decay profiles of **3PY** and **4PY** in the absence and presence of DCF to gain insights into the underlying quenching mechanism.

**Figure 8:**
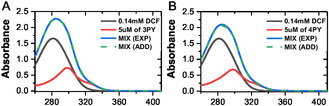
A) The absorbance here is shown for 0.14 mM DCF, 5 μM **3PY**, and the mix. B) The absorbance here is shown for 0.14 mM DCF, 5 μM, **4PY**, and the mix. ADD means the individual spectra were added manually, and EXP means the absorption was measured experimentally via a spectrometer, where the concentration of DCF and sensor was the same as the individual ones.

As shown in **Figure** **8**, the UV–vis absorption peak of DCF is observed at 280 nm, consistent with previously reported spectra,^[^ ^35^ ^]^ and no fluorescence is seen from the DCF as shown in Figure S21, Supporting Information, and **3PY**/**4PY** absorbance spectra exhibit a similar peak as the excitation spectra shown in Figure 5. To investigate ground‐state complexation or static quenching, we collected absorbance spectra for 0.14 mM DCF, 5 μM **3PY**/**4PY**, and their MIX. The term MIX (EXP) refers to absorbance recorded from a solution containing both 0.14 mM DCF and 5 μM **3PY**/**4PY**, whereas MIX (ADD) refers to the manually added absorbance spectra of free 0.14 mM DCF and 5 μM **3PY**/**4PY**. The complete overlap of MIX (ADD) and MIX (EXP) spectra indicates the absence of ground‐state complexation or static quenching. There is also no distortion in any absorbance and fluorescence band as shown in Figure 6A,B, and 8, which shows the absence of aggregation, thus aggregation‐based quenching is eliminated.

To gain information about electrostatic interactions between DCF and **3PY**/**4PY**, we measured the zeta potentials of each species. The zeta potential of the **3PY** and **4PY** was demonstrated to be −32 and −47 mV, respectively, and that of DCF is −51 mV. The higher magnitude (<−30 mV) of the zeta potential gives an indication of the potential stability of the colloidal system. If all the particles in suspension have a large negative or positive zeta potential, then they will tend to repel each other, and there will be no tendency for the particles to come together.^[^ ^36^ ^]^ Therefore, DCF has very little propensity to interact with **3PY** and **4PY** via electrostatic interaction due to both derivatives and DCF being highly negatively charged, complementing the absence of aggregation or static quenching.

Next, we considered FRET or dynamic quenching, both of which are distance‐dependent processes that require spectral overlap between the emission spectrum of the donor fluorophore (**3PY**/**4PY**) and the absorption (excitation) spectrum of the quencher/acceptor (DCF). However, as depicted in Figure 5, there is no significant spectral overlap between the absorption spectrum of DCF (maximum at ≈280 nm) and the emission spectrum of **3PY**/**4PY** (maximum at ≈387 nm), making FRET or dynamic quenching unlikely.

**Figure 9:**
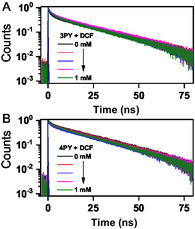
A) Decay profile for **3PY** in the presence and absence of DCF. B) Decay profile for **4PY** in the presence and absence of DCF. In both cases, the DCF concentration was varied from 0 to 1 mM. IRF here is shown in purple color, and the black color corresponds to the sample's decay curve in the absence of DCF. The decay was recorded at 404 nm (excited at 375 nm).

Fluorescence lifetime measurements of **3PY** and **4PY** were performed both in the presence and absence of DCF, with DCF concentrations varying from 0 to 1 mM, as illustrated in **Figure** **9**. The decay profiles were fitted with a multiexponential model, and amplitude‐weighted average lifetimes were calculated. The amplitude, corresponding time constants, and fit quality of the fluorescence lifetime data are detailed in the ESI Section 2, plotted in Figure S9 and S10, and tabulated in Table S1. The lifetimes of free **3PY** and **4PY** were measured to be 13 ns and 15.2 ns, respectively, using a two‐exponential fitting model. Upon the addition of 1 mM DCF, the lifetime of **3PY** decreased slightly from 13 ns to 12.5 ns, and that of **4PY** decreased from 15.2 ns to 14.2 ns. These modest reductions in lifetime could be due to (1) reabsorption or scattering effects at high analyte concentrations, which might indirectly affect the measurements, or (2) PET between the sensor and analyte, which can quench the excited state of the fluorophore via electron transfer, facilitated by suitable redox potentials and proximity. PET introduces a nonradiative relaxation pathway that competes with natural radiative and other nonradiative decay processes, such as internal conversion and intersystem crossing, thus shortening the overall excited‐state lifetime. However, the first explanation is unlikely since no anomalous spectral changes were observed in the absorption or fluorescence response of the sensor at high analyte concentrations. The slight reduction in lifetimes suggests that, if PET were occurring, its extent would be minimal. Therefore, the primary quenching mechanism is unlikely to be static, dynamic, FRET, electron transfer, or PET.

From careful examination of the absorption and excitation spectra in Figure 5 reveals a significant overlap between the absorption band of DCF and the fluorescence excitation wavelengths of the triphenylene derivatives. This overlap suggests that part of the excitation fluorescence of the derivatives could be absorbed and shielded by DCF, providing an opportunity for the IFE to occur, potentially as the primary quenching mechanism for **3PY** and **4PY**.

The overlap between the DCF absorption spectrum and the excitation spectra of **3PY** and **4PY** is substantial, at ≈60%. This percentage was calculated by dividing the area of overlap between DCF and **3PY**/**4PY** by the absorbance band area of DCF, revealing the extent of the absorbance band involved in overlap with the excitation band of **3PY**/**4PY**. The overlap is most pronounced for DCF at ≈60% (as presented in Figure S16, Supporting Information), and individual plots with the pharmaceutical compound's absorbance and excitation spectra of **3PY**/**4PY** are in the range of 15–40%, shown in Figure S17, Supporting Information. This extensive overlap is indicative of the selectivity of **3PY**/**4PY** for DCF among other pharmaceutical compounds.

To validate the role of IFE in fluorescence quenching, we corrected the fluorescence intensity using a specialized formula suitable for our spectrometer's horizontal slit geometry, differing from the conventional vertical slit configuration. The correction formula^[^ ^37^ ^]^ applied was

$$F_{\text{corr}}=F_{\text{obs}}\times 2.303A\left(x_{2}-x_{1}\right)/10^{{\text{−Ax}}_{1}}-10^{{\text{−Ax}}_{2}}\times 2.303A\left(y_{2}-y_{1}\right)/10^{{\text{−Ay}}_{1}}-10^{{\text{−Ay}}_{2}}$$

Herein, $F_{\text{corr}}$ is the corrected fluorescence intensity, $F_{\text{obs}}$ is the spectrally corrected fluorescence emission intensity from Cary, $x_{2}$ and $x_{1}$are the geometrical parameters for the detection beam, and $y_{2}$ and $y_{1}$ are the geometrical parameters for the incident beam. A is the absorbance at the excitation wavelength. Geometric parameters were determined using samples with minimal (tryptophan) and high reabsorption (Rhodamine B), with values chosen to align with the linear dependence between fluorescence and absorbance at the excitation wavelength. Additional details on IFE and methodology are provided in Section 4 of the ESI, and the calibration plot is shown in Figure S15, Supporting Information.

**Figure 10:**
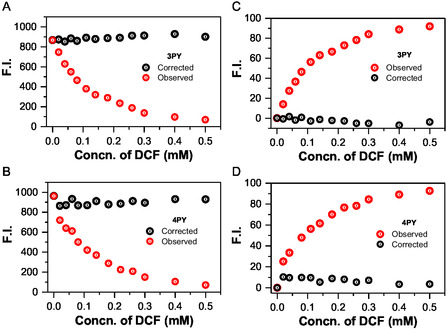
A,B) The I_obs_ and I_corr_ for the **3PY** and **4PY,** respectively. The corrected intensity with an increase in the concentration of DCF is shown in Black, and the observed intensity is shown in red. C,D) Suppression efficiency (*E*, %) is plotted for the **3PY** and **4PY,** respectively. The suppression efficiency of the corrected intensity is plotted in black, and the suppression efficiency for the observed fluorescence intensity is plotted in red.

The correction factor of the fluorescence IFE (I_corr_/I_obs_) and the observed (E_obs_) and corrected (E_corr_) fluorescence quenching efficiencies at various DCF concentrations were calculated and presented in Tables S2 and S3, Supporting Information. As shown in **Figure** **10**, the corrected fluorescence intensity of **3PY** is relatively constant, suggesting that the quenching is entirely due to IFE. However, in the case of **4PY**, the corrected fluorescence intensity decreases, and the suppression efficiency of corrected intensity increases (≈5%), possibly indicating a minor role of PET, consistent with the decay analysis. The lifetime decrease was 1 ns for **4PY** and 0.5 ns for **3PY**. The slight reduction in lifetime and increase in suppression efficiency in **4PY** suggest a small extent of PET, whereas in **3PY**, PET is negligible and not quantifiable from our experiments. Thus, it can be estimated that in **4PY**, quenching is 95% due to IFE and 5% due to PET, while in **3PY**, quenching is entirely through IFE. This aligns with the higher absorbance overlap of DCF with **3PY** compared to **4PY**, likely due to the broader excitation band of **4PY**, indicating that IFE is more pronounced in **3PY**.

To gain deeper insights into the quenching mechanisms involving IFE and PET, we utilized an electrochemical approach to study the interactions between the **3PY** and **4PY** sensors and the analyte DCF. Highest occupied molecular orbital (HOMO) and lowest unoccupied molecular orbital (LUMO) were estimated by the first oxidative and reductive onset of **3PY**, **4PY** and DCF taken from the CV. The detailed calculation and CV curve of **3PY**/**4PY**/DCF is shown in Figure S19, and the values are tabulated in Table S4, Supporting Information. Previously, spectroscopy and time‐resolved experimental analysis indicate that quenching predominantly occurs through the IFE, with a slight contribution from PET, specifically in the case of **4PY**. Based on the HOMO and LUMO levels, **3PY** has a HOMO of −5.84 eV and a LUMO of −3.74 eV, whereas DCF exhibits a HOMO of −4.88 eV and a LUMO of −3.74 eV. The alignment of these energy levels shows that the HOMO of **3PY** is more negative compared to that of DCF, and the LUMO levels are identical, thereby inhibiting any significant PET, leaving IFE as the sole quenching mechanism for **3PY**.

In contrast, **4PY** possesses a HOMO of −6.30 eV and a LUMO of −3.68 eV, positioning its LUMO slightly above that of DCF. This slight overlap facilitates a minimal PET process, accounting for about 5% of the quenching observed, alongside the predominant IFE. The small energy alignment difference between **4PY** and DCF allows for limited electron transfer. Therefore, while IFE remains the dominant quenching pathway for both **3PY** and **4PY**, the energy level configuration in **4PY** provides conditions conducive to a minor PET contribution, a phenomenon absent in **3PY**.

**Scheme 1:**
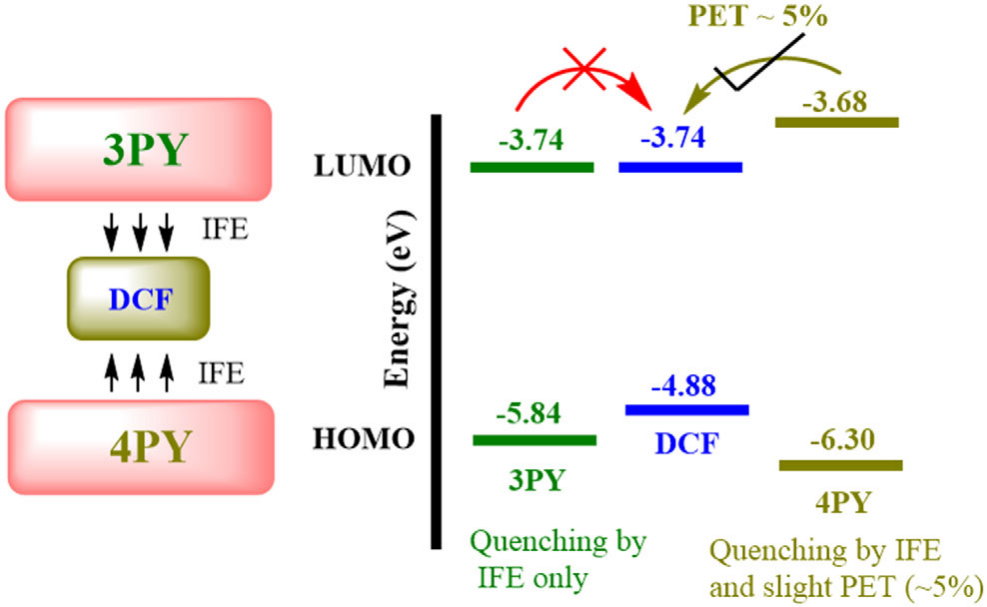
Plausible quenching mechanism of **3PY**/**4PY** in the presence of DCF.

DCF has previously been studied and acts as an acceptor molecule,^[^ ^38^, ^39^ ^]^ while **3PY/4PY** act as donor molecules. This distinction underscores the nuanced role of energy levels in dictating the quenching behavior of these sensor systems. Consequently, spectroscopic and electrochemical studies cumulatively suggest the key role of IFE in the quenching mechanism of **3PY/4PY**. At the same time, PET plays a minor role in the case of **4PY**, as shown in **Scheme** **1**. Note that Stern–Volmer analysis is not applicable further in our case because the primary quenching mechanism turns out to be IFE.

### 3.5 Detection in Real Water Samples

To further evaluate the feasibility of **3PY** and **4PY** for the rapid detection of DCF in real‐time samples, we collected water from Lake Jyväsjärvi in Jyväskylä, Finland. After centrifugation, lake water was filtered with a 0.2 μM membrane filter and spiked it with varying concentrations of DCF (20, 50, 80, and 250 μM). The results are shown in **Table** **1** and **2**. The spiked recoveries of DCF in different lake water samples ranged from 90.9–109.6%, with a SD ranging from 3–10%. SD was calculated from three replicate experiments. These findings demonstrated the method's potential utility for monitoring the DCF in real waters. The overall sensing capability is then compared with the other reported sensors, which show that the proposed sensors **3PY** and **4PY** have comparable or improved LOD as shown in **Table** **3**. The calibration plot of individual **3PY** and **4PY** sensors is shown in Figure S18, Supporting Information.

**Table 1:** Determination of DCF in lake water samples with sensor 3PY.

–	DCF spike [μM]	Found [μM]	Recovery [%]
Sensor 3PY	20	20.5 ± 1.8	102.4
	50	50.9 ± 3.1	101.7
	80	79.5 ± 7.4	99.4
	250	227.2 ± 20.5	90.9

**Table 2:** Determination of DCF in lake water samples with sensor 4PY.

–	DCF spike [μM]	Found [μM]	Recovery [%]
Sensor 4PY	20	21.9 ± 2.4	109.6
	50	54.3 ± 5.0	108.6
	80	82.4 ± 4.8	103
	250	252.3 ± 10	100.9

**Table 3:** Comparison of various reported sensors for the detection of DCF.

Material	Mechanism	Linear range [μM]	LOD [μM]	Ref
FMOF5	IFE	30–670	4.1	[40]
Cyclodextrins nanosponge	Static enhancement mechanism	1.0–33.0	0.92	[41]
Carbon quantum dots	electron transfer	5.0–300.0	2.33	[42]
MG‐Fe(β‐CD)	Potentiometric	–	1.2	[43]
**3PY**	**IFE**	**5–150**	**1.18**	**This work**
**4PY**		**5‐225**	**1.20**	

## 4 Conclusion

In conclusion, two pyridine‐modified triphenylene derivatives (**3PY** and **4PY**) were successfully synthesized for the sensitive quantitative detection of the DCF. These derivatives show strong emission intensity at 387 nm upon excitation at 300 nm. A range of commonly prescribed NSAIDs and hormones was also assessed, and the results indicated that **3PY** and **4PY** are selective for DCF over the other pharmaceuticals and hormones tested. These derivatives can serve as reliable and accurate fluorescent probes for DCF detection, based on quenching of their native fluorescent intensity due to IFE. The mechanism is further confirmed by the IFE corrections. The low detection limit and good linear range prove the utility of these fluorosensors for trace analysis of DCF. This work will provide impetus for synthesizing various fluorescent triphenylene derivatives and exploring their applications in sensing.

## Conflict of Interest

The authors declare no conflict of interest.

## Supporting information

Supplementary Material

## Data Availability

The data that support the findings of this study are available from the corresponding author upon reasonable request.
