# Supplementary material for: Diclofenac Detection via Inner‐Filter Effect Using Pyridine‐Modified Triphenylene
Source: Chempluschem. 2025 Jun 29;90(8):e202400687. doi: 10.1002/cplu.202400687 (PMC12352733; doi:10.1002/cplu.202400687)
Supplement: Supplementary file 1 — Supplementary Material [file CPLU-90-e202400687-s001.pdf]

Electronic Supplementary Information For

Diclofenac Detection via Inner-Filter Effect using Pyridine-Modified Triphenylene

Department of Chemistry, University of Jyväskylä, P. O. Box 35, FI-40014 Jyväskylä, Finland.

E-mail: matti.o.haukka@jyu.fi

Table of Contents

Section 1: Characterization of the Compounds

- Figure S1: ESI-QTOF-MS spectra measured for ligand 3py.
- Figure S2: ESI-QTOF-MS spectra measured for ligand 4py.
- Figure S3: 1H NMR of compound 3py in D2O+DCl.
- Figure S4: 13C NMR of compound 3py in D2O+DCl (CD3OD as an external reference).
- Figure S5: 1H NMR of compound 4py in D2O+DCl.
- Figure S6: 13C NMR of compound 4py in D2O+DCl (CD3OD as an external reference).
- Figure S7: FTIR of compound 3py.
- Figure S8: FTIR of compound 4py.

Section 2: TCSPC Decay Analysis of 3py and 4py in Presence and Absence of DCF

- Figure S9: Fluorescence decay analysis of 3py
- Figure S10: Fluorescence decay analysis of 4py
- Table S1: Tabulated data of the decay fitting with the multiexponential function.

Section 3: Steady State Spectra and Parameter Analysis of 3py and 4py in Presence and Absence of DCF

- Figure S11: Molar extinction coefficient of 3PY and 4PY
- Figure S12: Absorbance and fluorescence plot of 3PY/4PY and the standard reference compound, Quinine sulfate (QS), for doing the relative quantum yield calculation.
- Figure S13: Fluorescence quenching and fluorescence emission spectra of 3PY/4PY in presence of different drugs. Excitation dependent fluorescence spectra of 3PY/4PY.

Section 4: Inner Filter Effect Correction and Methodology

- Figure S14: Schematic depiction of a top view of the cuvette with the fluorescence observation field (FOF)

Section 4.1: Experimental Procedure

- Figure S15: The fluorescence intensity vs absorbance at excitation wavelength profile for Rhodamine (A) and tryptophan (B).
- Table S2: Inner Filter analysis of 3PY in presence of DCF
- Table S3: Inner Filter analysis of 4PY in presence of DCF

Section 5: Area Overlaps Comparison In between Pharmaceutical Samples

- Figure S16: Bar graph showing area of overlap of pharmaceuticals with area of excitation spectra band of 3PY and 4PY
- Figure S17: Pharmaceutical compounds absorbance spectra with the excitation spectra of 3PY and 4PY.

Section 7: Sensing in Real Samples

- Figure S18: A and C are the calibration plot for 3PY and 4PY respectively. The calibration plot for 3PY is multiplied by a constant 1.73. B and D is the fluorescence intensity profile monitored at 400 nm in the lake and deionized water.

Section 8: Electrochemical studies

- Figure S19: Electrochemical response of 3PY, 4PY, DCF and Ferrocene
- Table S4: Electrochemical parameter of 3PY/4PY/DCF.

Section 9: Effect of alkali, alkaline earth, and heavy metal ions with 3PY and 4PY.

- Figure S20: Effect of alkali, alkaline earth, and heavy metal ions with 3PY and 4PY. (Concentration of probe and cations is 5 μM and 500 μM respectively.)

Section 10: Fluorescent spectra of DCF and standard deviation of the measured fluorescence intensities at concentrations close to the calculated LOD.

- Figure S21: Emission spectra of DCF (2 mM) in ethanol (excitation 280 nm).
- Figure S22: A) Fluorescent spectra of 3PY at different concentrations starting from 50nM to 5 μM in DMSO at (λex= 300nm). B) Normalized fluorescent spectra of 3PY (Fig.A). C) Fluorescent spectra of 4PY at different concentrations starting from 50nM to 5 μM in DMSO at (λex= 300nm). D) Normalized fluorescent spectra of 4PY (Fig.C). E) Fluorescent spectra of 3PY (5 μM) with different concentrations of DCF starting from 1 μM to 10 μM in DMSO at (λex= 300nm). F) Fluorescent spectra of 4PY (5 μM) with different concentrations of DCF starting from 1 μM to 10 μM in DMSO at (λex= 300nm). G) Standard deviation of the measured fluorescence intensities at DCF concentrations close to the calculated LOD (1 μM to 10 μM) in DMSO with 5 μM of probe excited at 300nm (seven measurements).

Section 1: Characterization of the compounds

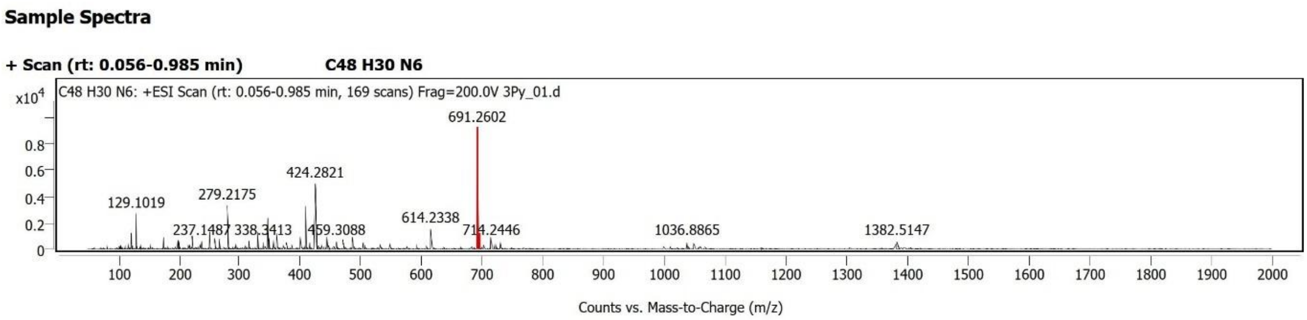

Figure S1: *ESI-QTOF-MS spectra measured for ligand 3py.*

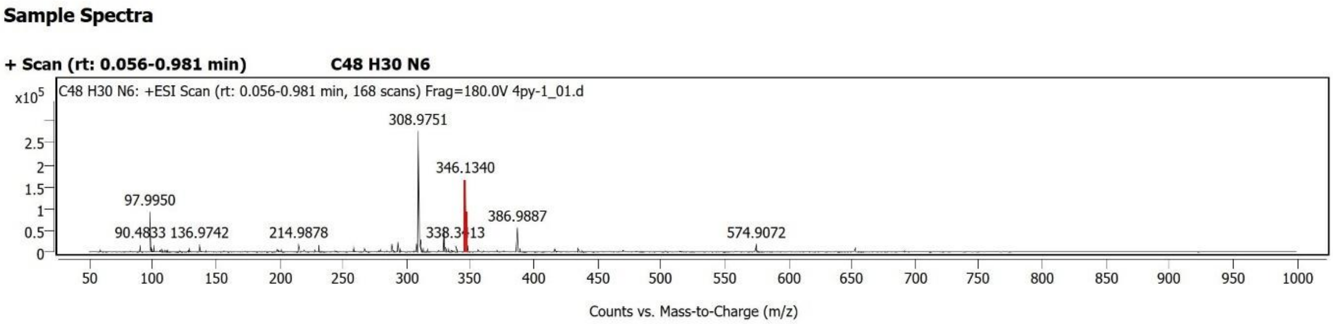

Figure S2: *ESI-QTOF-MS spectra measured for ligand 4py.*

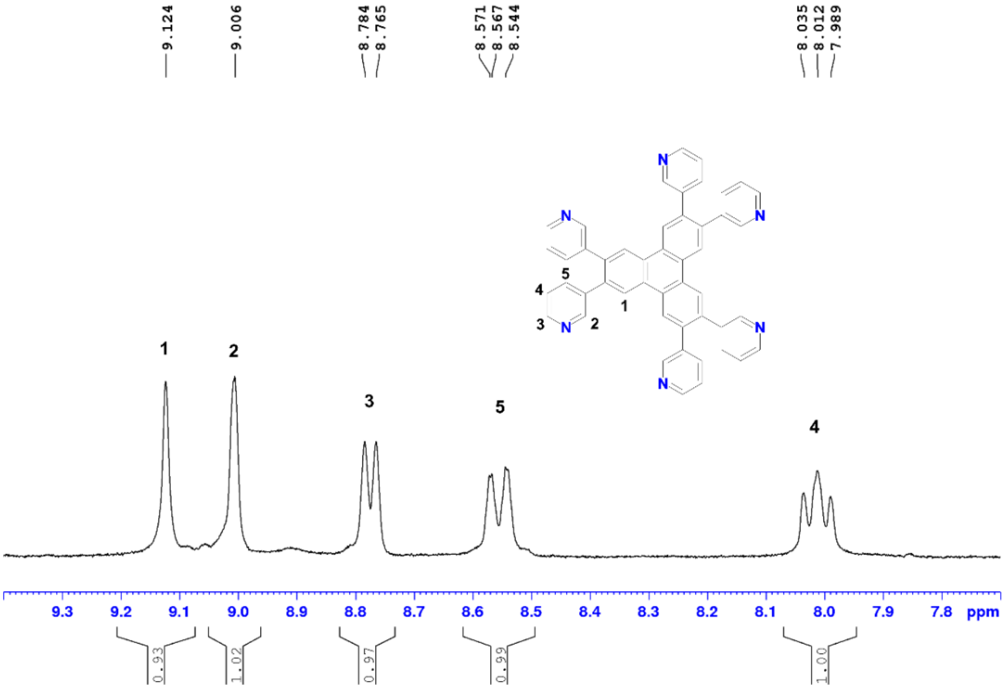

Figure S3: *^{1}H NMR of compound 3py in D_2O+DCl.*

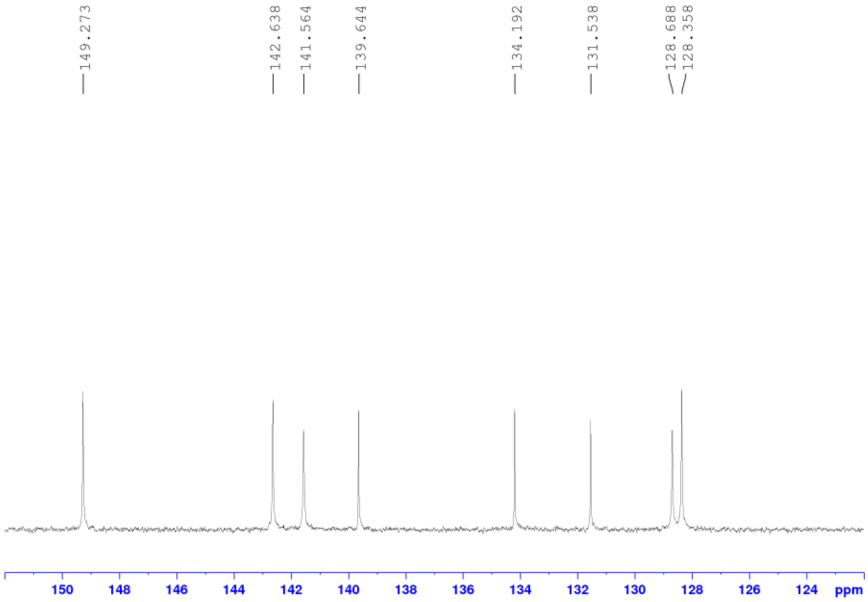

Figure S4: *^{13}C NMR of compound 3py in D_2O+DCl (CD_3OD as an external reference).*

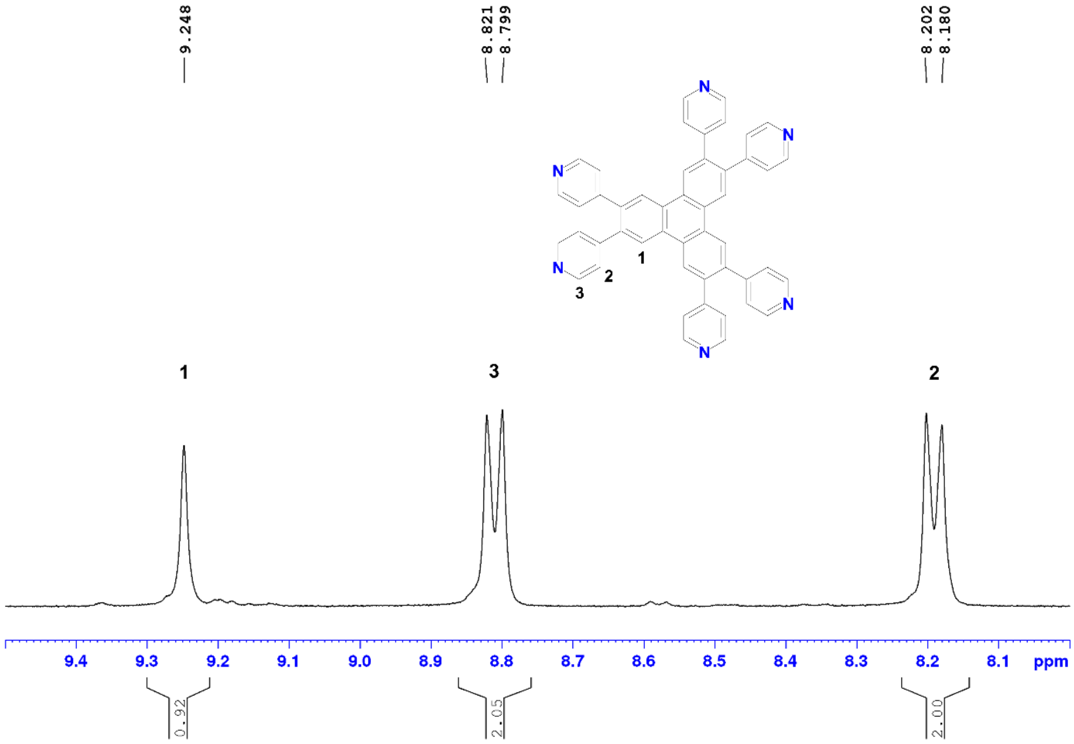

Figure S5: *^{1}H NMR of compound 4py in D_2O+DCl.*

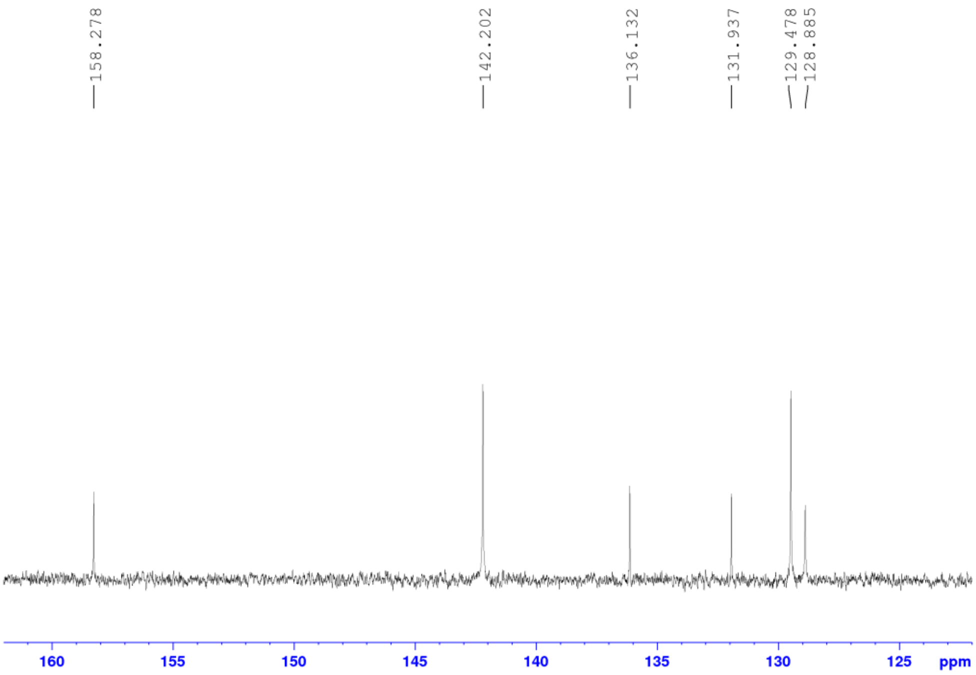

Figure S6: *^{13}C NMR of compound 4py in D_2O+DCl (CD_3OD as an external reference).*

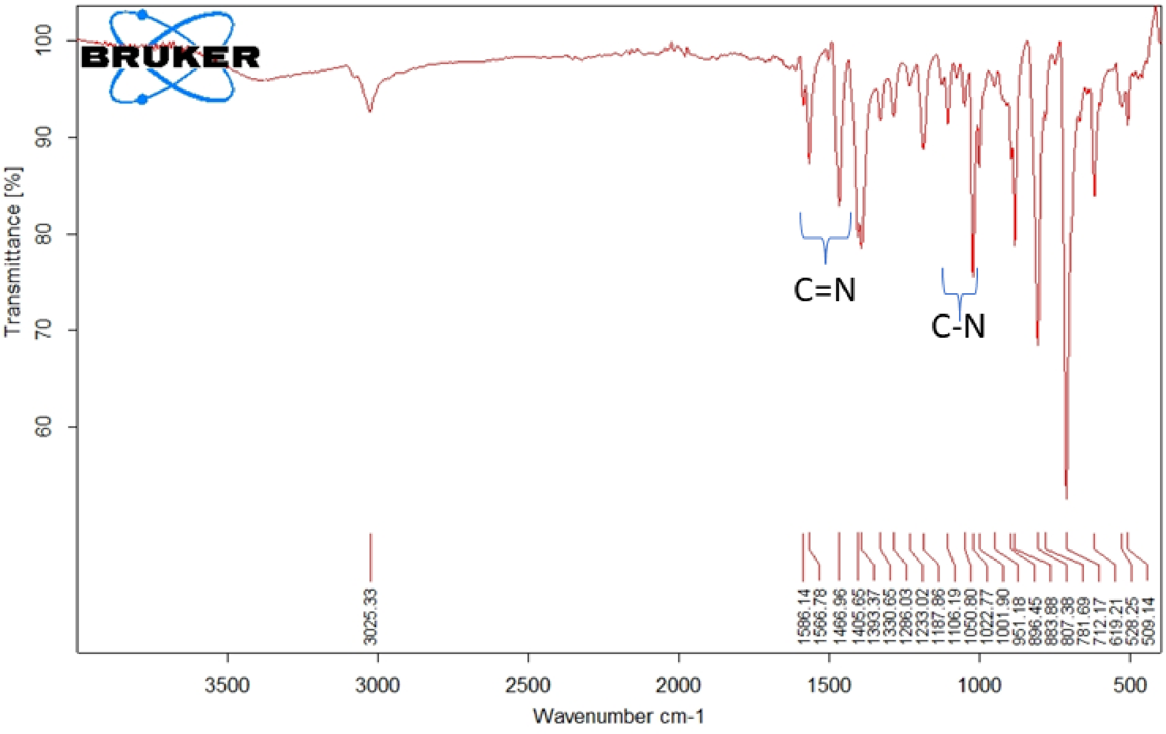

Figure S7: *FTIR of compound 3py.*

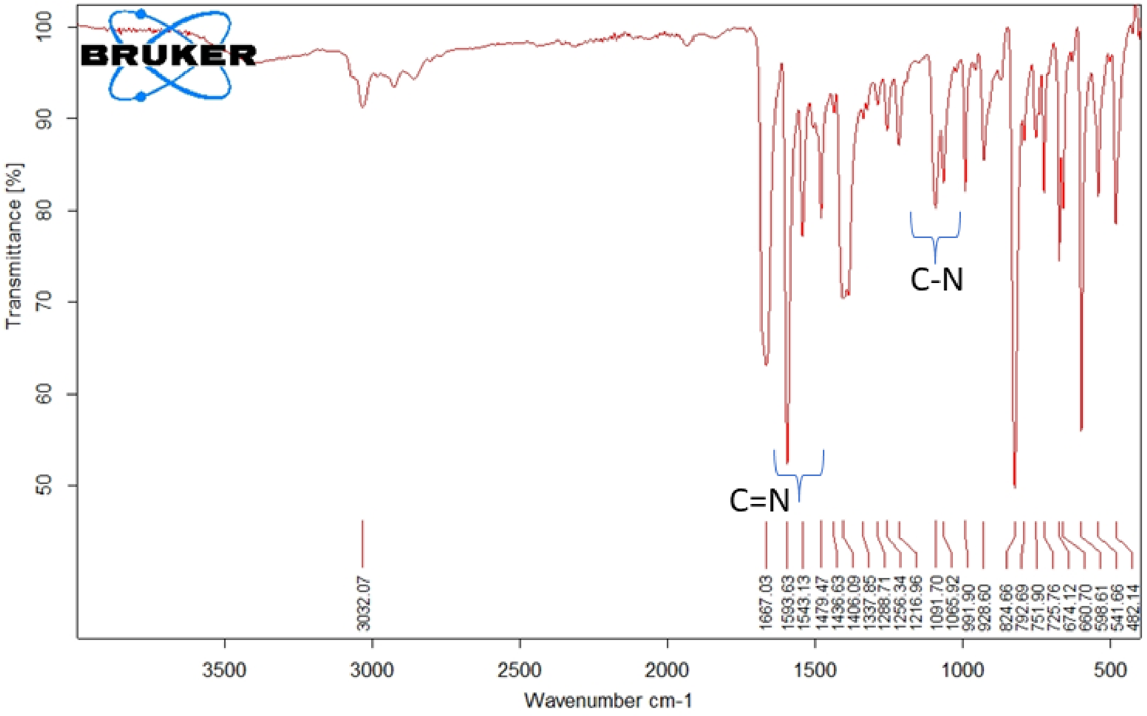

Figure S8: *FTIR of compound 4py.*

Section 2: TCSPC decay analysis of 3py and 4py in presence and absence of DCF

In our experimental methodology, we employed time-resolved optical spectroscopy, utilizing the PicoQuant HydraHarp 400 system—a commercially available tool designed for time-correlated single photon counting (TCSPC) data acquisition. The excitation source consisted of a PicoQuant PDL 800-D pulsed diode laser driver and a diode laser head (LDH-PC-375) emitting light at 375 nm wavelength (with a spectral full width at half maximum of 4 nm). The excitation pulses were consistently generated at a repetition rate of 8 MHz for all measurements. Additionally, we adjusted the laser power for each sample to achieve an average count rate at the detector equivalent to 0.8% of the excitation rate.

To assess temporal resolution, we employed a scattering sample, which yielded an estimated value of approximately 148 ps for 375 nm (corresponding to the full width at half maximum of the instrument response function). All measurements were conducted under standard room temperature and ambient conditions, with sample volumes fixed at 1.5 ml.

For data analysis, we deconvoluted the decay traces using the instrument response function (IRF) measured via scattering from silica at the excitation wavelength. Our custom script employed an initial guess of three exponential components, and we iteratively optimized the fit to obtain the best chi-square value. Additionally, we calculated weighted residuals using the equation (Raw-Fit)/(Raw^0.5), where ‘raw’ represents the non-fitted decay traces and ‘fit’ corresponds to the fitted data with multiexponential components. The bin width of traces recorded were 16 ps.

The decay spectra were fitted by the two decay components. The formula is shown below, here α_i is the fractional amplitude of that component, I(t) is the fluorescence intensity as a function of time, t and τ_i is the fluorescence lifetime of the ith decay component. Here, n is equal to 2.

$$I(t) = I_0 \sum_{i=1}^{n} \alpha_i \exp\left(\frac{-t}{\tau_i}\right) \quad \text{.............................(1)}$$

Table S1: *Samples and their associated fitting output parameters are tabulate here. We have a total of 10 sets of samples and all of them were fitted with the two decay components and one rising component. The fitting quality parameter is also mentioned as the* χ_R^2.

Sample	α_1	τ_1 (ns)	α_2	τ_2 (ns)	χ_R^2	τ_{avg} (ns)[A]
4PY	0.32	1.3 (0.3)	0.68	21.8 (0.2)	1.4	15.2 (0.2)
4PY + 0.16mM DCF	0.32	1.4 (0.3)	0.68	21.6 (0.2)	1.4	15.1 (0.2)
4PY + 0.28mM DCF	0.35	1.4 (0.2)	0.65	21.6 (0.2)	1.4	14.5 (0.2)
4PY + 0.52mM DCF	0.33	1.7 (0.3)	0.67	21.5 (0.2)	1.4	15 (0.2)
4PY + 1mM DCF	0.35	1.7 (0.3)	0.65	21 (0.2)	1.3	14.2 (0.2)
3PY	0.47	1.6 (0.2)	0.53	23.1 (0.3)	1.3	13 (0.3)
3PY + 0.16mM DCF	0.46	1.8 (0.2)	0.54	22.8 (0.3)	1.4	13.1 (0.3)
3PY + 0.28mM DCF	0.47	1.9 (0.2)	0.53	22.5 (0.3)	1.4	12.8 (0.3)
3PY + 0.52mM DCF	0.47	1.9 (0.2)	0.53	22.4 (0.2)	1.2	12.8 (0.2)
3PY + 1mM DCF	0.49	2.3 (0.2)	0.51	22.2 (0.3)	1.3	12.5 (0.3)

[A]*The amplitude weighted average lifetime was calculates using* $\tau_{avg}(ns) = \frac{\alpha_i\tau_1+\alpha_2\tau_2}{a_1+\alpha_2}$*, only accounting the decay component.*

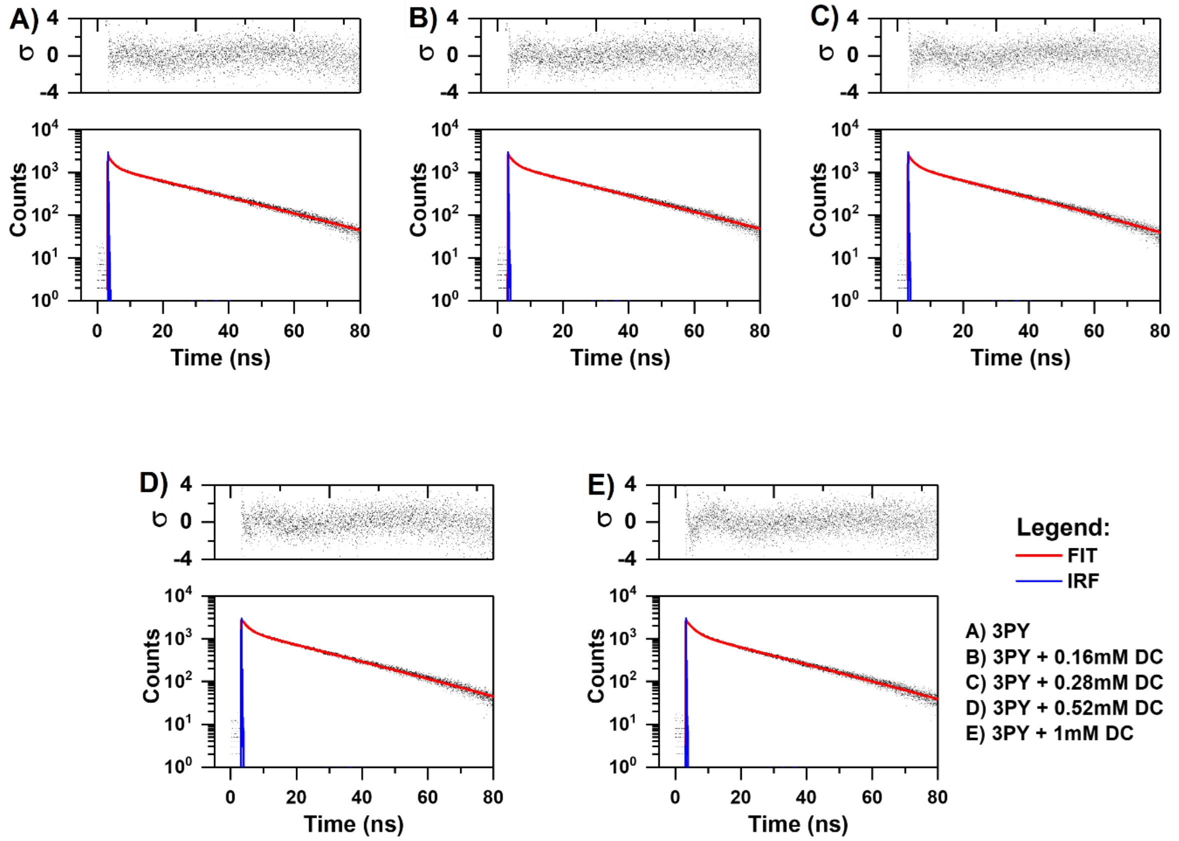

Figure S9: *Decay curves and the residuals plotted for 3PY in presence and absence of DCF. The decay was recorded at 404 nm wavelength and the sample was excited using a 375 nm laser diode. The concentration of 3PY sensor was 20uM and the DCF was varied from 0 to 1mM.*

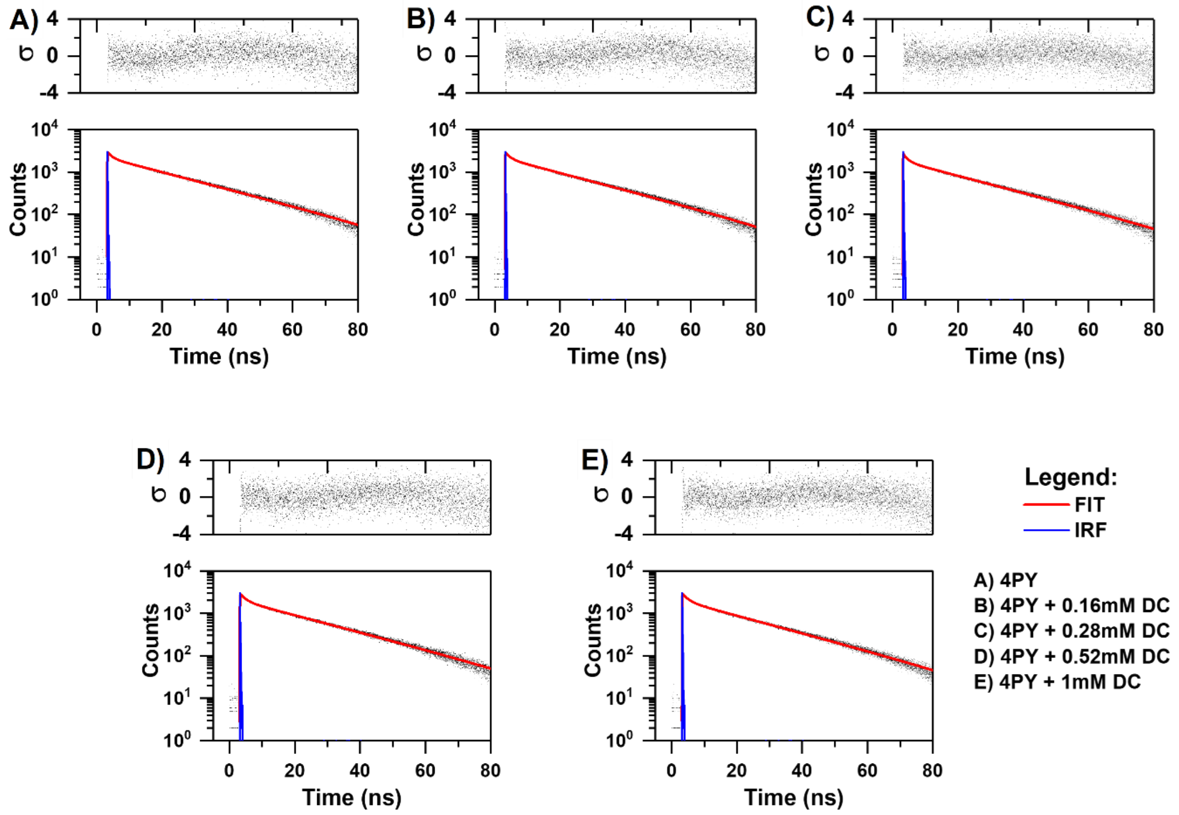

Figure S10: *Decay curves and the residuals plotted for 4PY in presence and absence of DCF. The concentration of 4PY sensor was 20uM and the DCF was varied from 0 to 1mM. The decay was recorded at 404 nm wavelength and the sample was excited using a 375 nm laser diode.*

Section 3: Steady state spectra and parameter analysis of 3PY and 4PY in presence and absence of DCF

First, we calculated the molar extinction coefficient for 3PY and 4PY sample as shown in the figure S11. The synthesized probes, 3PY and 4PY, exhibit good solubility in DMSO, allowing us to easily achieve the 50 µM concentration and then diluted it as per requirement. Here, the concentration of 3PY differs from 1.5625uM to 25uM and for 4PY the concentration range is from 3.125uM to 25uM. The plot of absorption value vs concentration will give us $\varepsilon \times l$ where ε is the molar absorption value and l is the path length which was 1 cm in our case. So, the slope will be equal to ε after converting uM to M. Slope of 0.132 will give us 132000 ± 2000 $M^{-1}cm^{-1}$ and slope of 0.120 will give us 120000 ± 2000 $M^{-1}cm^{-1}$.

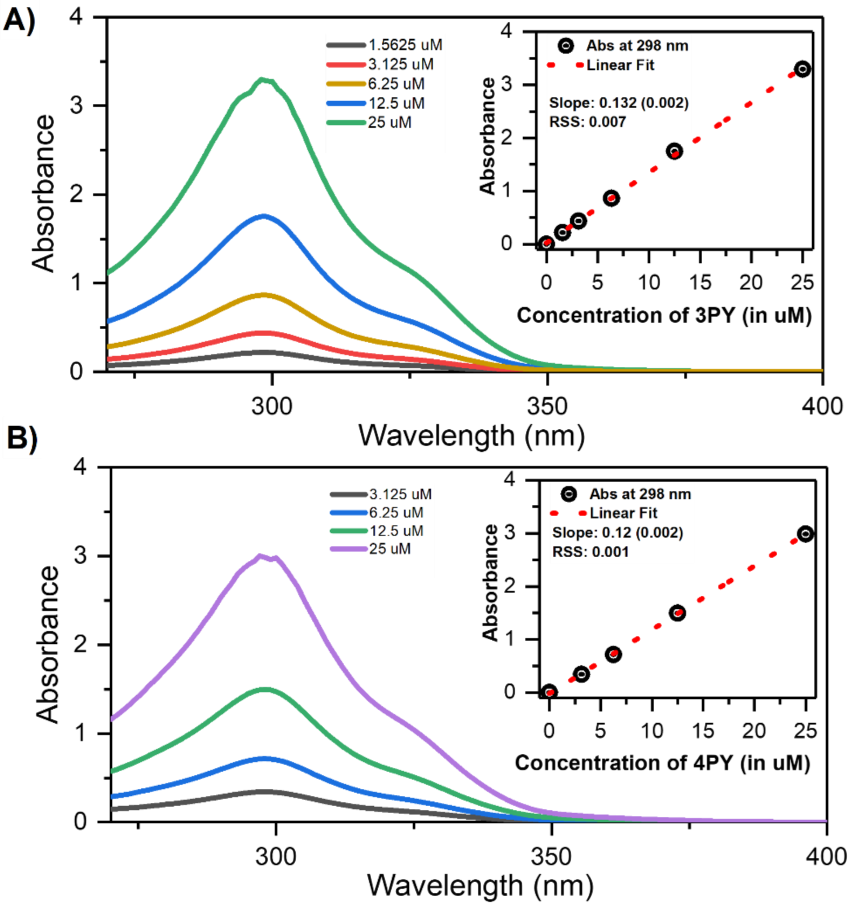

Figure S11: *Extinction coefficient calculate with iteration of sample's state absorption spectra at different concentrations (in DMSO). The figure shows the UV-Vis absorption spectra at different concentrations and the inset figure is the linear fitting of the absorption value at 298 nm vs concentration plot.*

Quantum yield estimation

The quantum yield of 3PY/4PY (ϕ_{PY}) was estimated using quinine sulfate in 0.105 M $HClO_4$ (quantum yield, 0.59 ± 0.04) as a reference fluorescence compound [1]. The results were calculated according to equation 1:

$$\emptyset_{CD} = \frac{f_{std}}{f_{CD}} \times \frac{F_{CD}}{F_{std}} \times \frac{n_{CD}^2}{n_{std}^2} \times \emptyset_{std} \qquad \ldots\ldots\ldots\ldots\ldots\ldots\ldots(2)$$

where, ϕ_{std} is the known quantum yield of the standard compound, F_{CD} and F_{std} stands for the integral photon flux of 3PY/4PY and quinine sulfate (standard) and calculated as according equation 2.

$$\mathrm{F} = \int_{em} I_C \cdot d\lambda_{em} \qquad \ldots\ldots\ldots\ldots\ldots\ldots\ldots(3)$$

Where I_C, is the corrected fluorescence spectra involving the spectral correction and blank correction. The absorbance of standard and 3PY/4PY was at 300 nm and the slit width of absorption spectrometer and excitation slit width of fluorescence spectrometer was fixed at 2.5 nm.

f_{std} and f_{CD} are the absorption factor of the standard and 3PY/4PY at the λ_{ex}= 300 nm. Here absorption factor was taken as the absorbance value at the excitation wavelength since IFE was corrected (described in next section).

n^2_{CD} & n^2_{std} are the refractive index of water. PL emission spectra of 3PY/4PY solution were measured at λ_{ex}= 300 nm, and the calculated percentage quantum yield was 0.223 and 0.256 for 3PY and 4PY respectively.

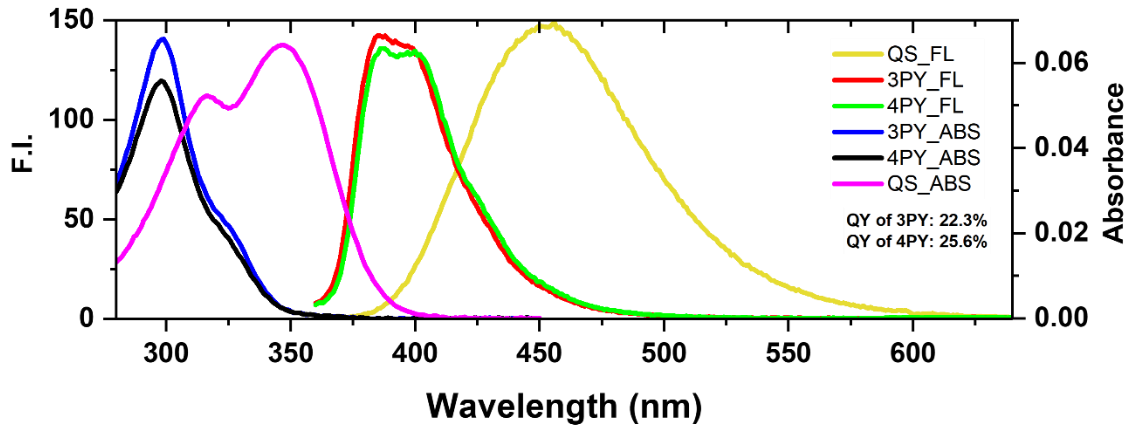

Figure S12: *Absorbance and fluorescence plot of 3PY/4PY and the standard reference compound, Quinine sulfate (QS), for doing the relative quantum yield calculation. Yellow, red and green correspond to the fluorescence plot of QS, 3PY and 4PY respectively. Blue, black and pink correspond to the absorbance plot of 3PY, 4PY and QS respectively. The absorbance values are on right and fluorescence on left.*

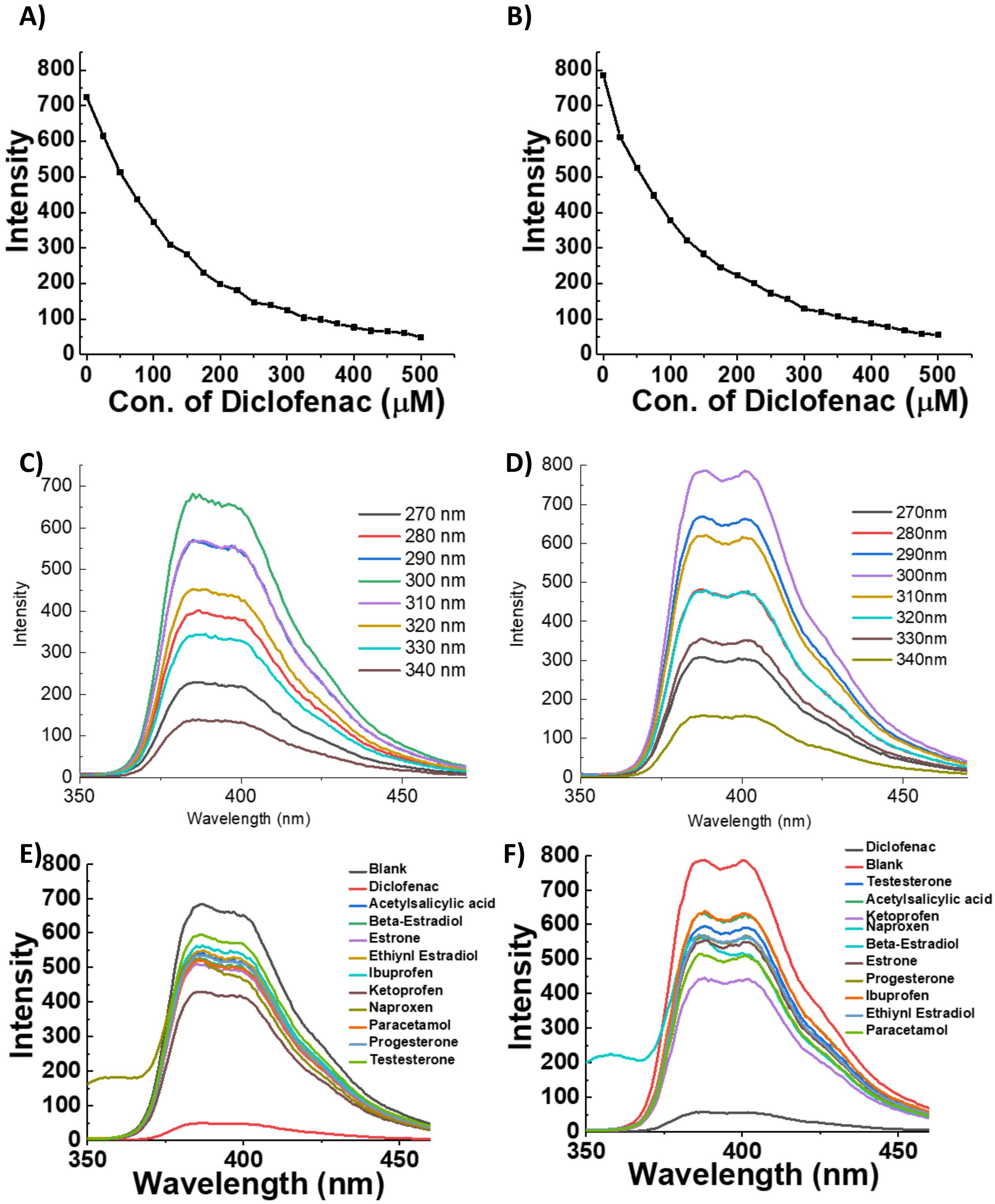

Figure S13: *A and B represents the quenching of fluorescence intensity of 3PY (5µM) and 4PY (5µM) with different concentration of DCF respectively (0-500µM). C and D represents the excitation dependent emission spectra of 3PY and 4PY respectively. E and F represents the emission spectra of 3py and 4py in presence of various commonly prescribed drugs and hormones (0-500uM) respectively.*

Section 4: Background for inner filter effect correction and methodology

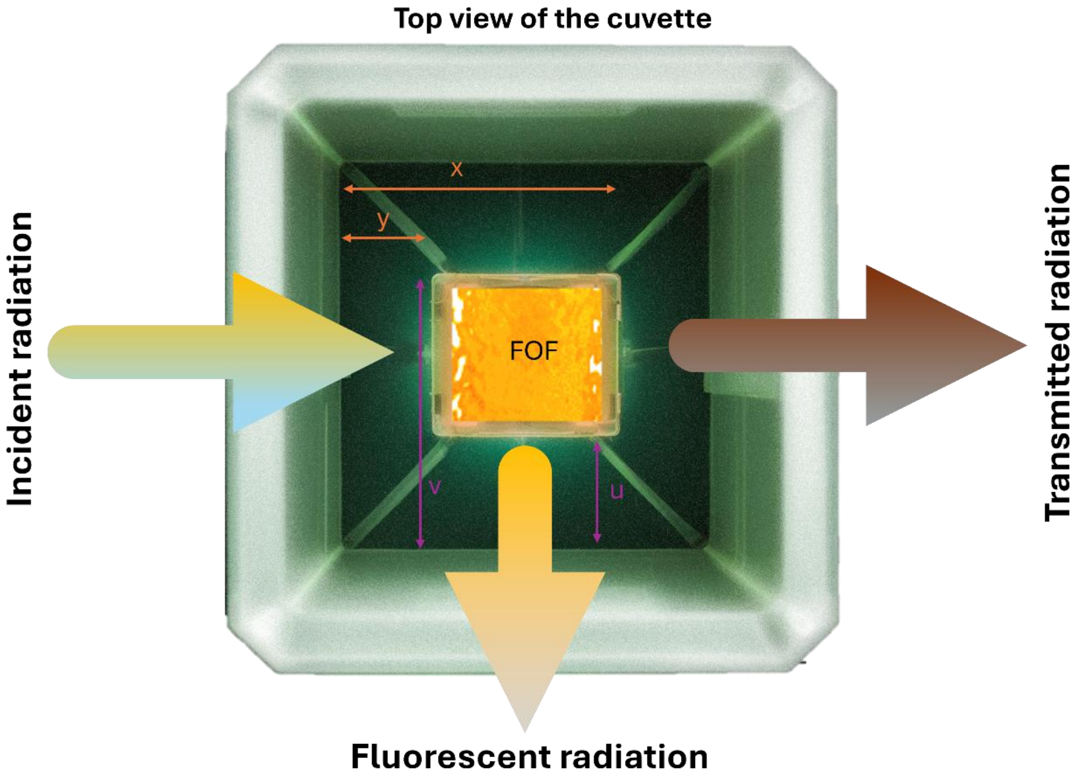

Figure S14: Schematic depiction of a top view of the cuvette with the fluorescence observation field (FOF) in between. u, v, x, and y are the FOF parameters of the cell. Here we are using the term FOF, which is common in most of the correction methods proposed. However, researchers have used other terms like "observation window", "interrogation zone" and "influence zone". It is important to note that the FOF is expanding symmetrically outwards from the center of the standard 1 cm path length cuvette cell.

In conventional right-angle fluorescence observation, the detection unit captures fluorescence radiation from only a limited central region of the sample compartment (cuvette cell), referred to as the fluorescence observation field (FOF, Fig. S14). Notably, the FOF is centered within the standard 1 cm path length cuvette and symmetrically expands from this center [11]. Both the excitation and emission light are subject to attenuation as they enter and exit the FOF, respectively. The extent of this attenuation depends on the concentration of the solution at the excitation (λ_{ex}) and emission wavelengths (λ_{em}), as well as the dimensions of the FOF. According to Lambert-Beer's law, light intensity decreases exponentially within the fluorescence cell, as described by Eq 4 for radiation intensity and Eq 5 for percentage transmission [7-11].

$$A = log\left(\frac{I_0}{I}\right) = \varepsilon \cdot C \cdot l \qquad I = Io * 10^{\varepsilon.c.l} \qquad \dots\dots\dots\dots\dots\dots\dots.(4)$$

$$A = log\left(\frac{1}{T}\right) = log\left(\frac{100}{\%T}\right)$$
$$A = 2 - log(\%T) \qquad \text{--------------------------------(5)}$$
$$10^{(2-A)} = \%T$$

In these equations, A represents absorbance, I_0 and I denote the intensity of excitation radiation before and after passing through the cell, respectively, T is transmittance, ε is the molar extinction coefficient of the analyte, c is concentration, and l is the path length of the measuring beam in the sample. HORIBA previously reported that this well-known inner filter effect (IFE) is influenced by sample absorption and instrument geometry, often becoming significant even in samples with low absorption (an error of about 8% at an absorbance of 0.06 in a 1 cm square cell)[5]. Additionally, Slageren et al have done a detailed analysis of various factors influencing the fluorescence response in reflection type, perpendicular type, and transmission type fluorescence measurement geometry and verified that true linear relation between I_f (fluorescence intensity) and c (concentration) never exists [4].

The fluorescence emission intensity (I_f, Eq 6) is directly proportional to the quantum yield of the fluorophore (ϕ), its molar extinction coefficient at the excitation wavelength (ε), and the intensity of the excitation radiation (I_{ex}). However, an apparent quenching of fluorescence can occur due to the attenuation of the excitation beam, known as the primary inner filter effect (IFE), and/or reabsorption of the emitted radiation by the absorber/quencher, known as the secondary IFE. The IFE is a radiative energy transfer phenomenon where the excitation and/or emission energy of the fluorophore is absorbed by the quencher, leading to an initial increase in fluorescence intensity, which then decreases as the sample concentration increases.

$$I_f \ \alpha \ \phi \times \varepsilon \times I_{ex}$$
$$I_f \ \alpha \ \phi \times \varepsilon \times I_0 \times 10^{-\varepsilon c \cdot l} \qquad \text{-------------------------------(6)}$$

When multiple absorbing species are present in the sample matrix, accurate quantitative estimation of such multi-component systems can only be achieved after correcting for the observed fluorescence intensity due to IFE. Various correction methods have been developed over time as our understanding of the factors' influencing IFE has improved. Note that there are always some common assumptions in correcting IFE [3]. Starting with the pioneering work of Parker in the 1960s, several procedures have been proposed for recovering the corrected luminescence intensity of samples affected by IFE. The general correction strategy is based on Eq 7 [5].

$$F_{corr} = f_{sec} * f_{prim} * F_{obs} \qquad \text{----------------------------------(7)}$$

In Eq 7, Fobs represents the observed fluorescence intensity, F_{corr} is the corrected fluorescence intensity, f_{prim} is the correction factor for primary IFE, and f_{sec} is the correction factor for secondary IFE. The simplest method for correcting IFE, mentioned by Lakowicz, is described in Eq 8. However, this approach is approximate, assuming that emission radiation is observed at the center of the 1 cm path-length cuvette cell, with a fluorescence observation point (lp) of 0.5. This assumption weakens the correction function compared to others [9].

$$F_{corr} = F_{obs} * 10^{(A\lambda ex + A\lambda em)/2} \qquad \text{----------------------------------(8)}$$

In Eq 8, $A_{\lambda ex}$ and $A_{\lambda em}$ represent the optical density of the solution at the excitation and emission wavelengths of the fluorophore, respectively. Kubista et al. empirically derived a correction function for primary IFE specific to their instrumental setup, expressed in Eq 9. In this equation, l_p was empirically determined to be 0.56 in their study, differing from the value assumed in Lakowicz's model. Kubista's correction function is widely adopted due to its applicability to a broader range of spectrometers. [8]

$$F_{corr} = F_{obs} \times 10^{(A\lambda_{ex} * lp)} \quad \text{-------------------------------------(9)}$$

Parker and Barnes proposed that IFE depends on the geometry of the spectrofluorometer. For parallel excitation beams, they suggested a correction method for primary IFE [7]. When absorbance is high, the correction factor becomes significant, and the accuracy of this factor depends on precise optical density measurements. Their correction factor for primary IFE and secondary IFE [2] is given in Eq 9.

$$f_P = \frac{F_o}{F} = \frac{2.303A(x_2 - x_1)}{10^{-Ax_1} - 10^{-Ax_2}} \quad \text{---(10)}$$

$$f_s = \frac{F_o}{F} = \frac{2.303A(y_2 - y_1)}{10^{-Ay_1} - 10^{-Ay_2}} \quad \text{--(11)}$$

Holland et al. proposed a model that corrects for the attenuation of excitation light due to chromophores in the sample matrix. In a subsequent study, they considered the FOF dimensions and proposed a correction factor for secondary IFE (Eq 11). This factor, when combined with the primary IFE correction factor from Eq 7, results in the total corrected fluorescence intensity (Eq 10), accounting for both primary and secondary IFE. The utility of Eq 12 has been validated in correcting IFE for organic dye solutions at higher optical densities as reported by Kasperek et al [3].

$$F_{corr} = F_{obs} \times \frac{2.303A(x_2 - x_1)}{10^{-Ax_1} - 10^{-Ax_2}} \times \frac{2.303A(y_2 - y_1)}{10^{-Ay_1} - 10^{-Ay_2}} \quad \text{----------------------------(12)}$$

Section 4.1: Experimental Procedure

Absorption spectra were recorded using a Cary 500 spectrometer (Agilent, Australia) with 10 mm quartz cells. Fluorescence measurements were conducted using a Cary Eclipse fluorimeter (Agilent, Australia) with a 10 × 10 mm quartz cell and right-angle geometry. The emission slit was set to 5 nm, and the excitation slit to 2.5 nm, with PMT detector voltage of 600 V. The total fluorescence (TF), represented by the area under the spectrum, was used as the measure of fluorescence intensity.

The beam width parameters (y2 – y1) for the incident beam and (x2 - x1) for the detection beam) were calculated in two parts: the first using a sample with minimal reabsorption (tryptophan) and the second using a sample with high reabsorption (Rhodamine B). The MATLAB algorithm first corrects the fluorescence spectra for instrument sensitivity, inputting the absorbance at the excitation wavelength (A_{ex}) and sets of x1 and x2 parameters. The fluorescence spectra are then corrected for primary IFE using the f_p function, and for secondary IFE using the f_s function with initial y1 and y2 values. The process is repeated for different sets of x1 and x2 parameters, with the final set chosen based on the highest R2 coefficient for the linear dependence between TF and A_{ex}. Similarly, for y1 and y2 determination, the parameters with the lowest mean squared error (MSE) for normalized corrected fluorescence spectra are selected.

After, computing x1, x2, y1 and y2 and tested with the Rhodamine and tryptophan sample as shown in figure below. The width parameters and the overall process was further applied on the 3PY and 4PY samples.

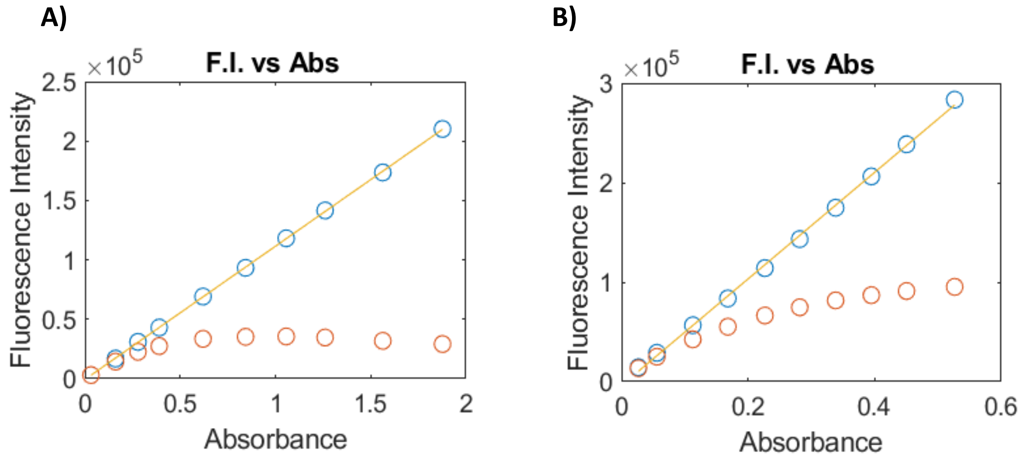

Figure S15: *The fluorescence intensity vs absorbance at excitation wavelength profile for Rhodamine (A) and tryptophan (B). The red color denotes the IFE uncorrected spectra and blue color denotes the IFE corrected spectra. These two samples are the calibration plot for further IFE correction.*

Table S2: Inner Filter analysis of 3PY in presence of DCF

DCF conc. (mM)	I_{corr}	I_{obs}	I_{corr} /I_{obs}	I_{corr} /Io_{corr}	I_{obs}/Io_{obs}	E_{obs}	E_{corr}
0	866.9	866.6	1.0003	1.0	1.00	0	0
0.02	872.7	745.8	1.1701	1.0	0.91	13.9	0
0.04	852.7	628.7	1.3562	1.0	0.74	27.4	2
0.06	883.6	549.5	1.6079	1.0	0.69	36.6	-1
0.08	860.1	464.5	1.8513	1.0	0.58	46.4	1
0.11	891.3	379.4	2.3490	1.0	0.49	56.2	-2
0.14	878.0	320.3	2.7407	1.0	0.37	63.0	-1
0.18	886.9	289.3	3.0648	1.0	0.33	66.6	-2
0.22	890.6	234.6	3.7959	1.0	0.27	72.9	-2
0.26	911.7	188.4	4.8371	1.1	0.22	78.3	-5
0.3	912.1	137.5	6.6298	1.1	0.16	84.1	-5
0.4	928.3	96.9	9.5741	1.1	0.11	88.8	-7
0.5	899.9	69.6	12.921	1.0	0.08	92.0	-3

Table S3: Inner Filter analysis of 4PY in presence of DCF

DCF conc. (mM)	I_{corr}	I_{obs}	I_{corr} /I_{obs}	I_{corr} /Io_{corr}	I_{obs}/Io_{obs}	E_{obs}	E_{corr}
0	962.5	962.1	1.0	1.00	1	0	0
0.02	863.6	720.3	1.2	0.89	0.75	25.1	10.3
0.04	870.4	640.1	1.4	0.90	0.67	33.5	9.6
0.06	932.2	611.5	1.5	0.97	0.64	36.4	3.1
0.08	867.7	501.1	1.7	0.90	0.52	47.9	9.8
0.11	869.8	420.5	2.1	0.90	0.44	56.3	9.6
0.14	910.7	369.9	2.5	0.95	0.38	61.5	5.4
0.18	878.1	287.7	3.1	0.91	0.30	70.1	8.8
0.22	885.2	224.7	3.9	0.92	0.23	76.6	8.0
0.26	911.5	208.3	4.6	1.00	0.21	78.3	5.3
0.3	894.1	149.5	5.9	0.93	0.15	84.5	7.1
0.4	930.8	105.1	8.8	0.97	0.11	89.1	3.3
0.5	928.8	70.6	13.7	0.96	0.07	92.6	3.5

Section 5: Area overlaps comparison in between pharmaceutical samples

The area overlap of different pharmaceutical samples (PS) was analyzed and tabulated in the figures. The percentage of area of absorbance band of PS overlapped was calculated by using the area overlap divided by individual area of absorbance band of PS. The are overlap was calculated using a MATLAB script. Here is a brief description about the calculation of area related figures. The script is designed to analyze absorbance data from two sources over a specified wavelength range. It interpolates the data to a common wavelength axis, calculates

the areas under the absorbance curves, and fits Gaussian functions to the data around the peak absorbance. By aligning the absorbance data to a common wavelength axis, the script ensures accurate comparison. It computes the area under each absorbance curve and the overlap area using the trapezoidal rule. Additionally, Gaussian functions are fitted to each absorbance dataset around the peak maxima, and the areas under these fits are calculated, including the complete Gaussian areas. The results, including the areas for each dataset, their overlaps, and Gaussian fit parameters, are displayed in the below figures.

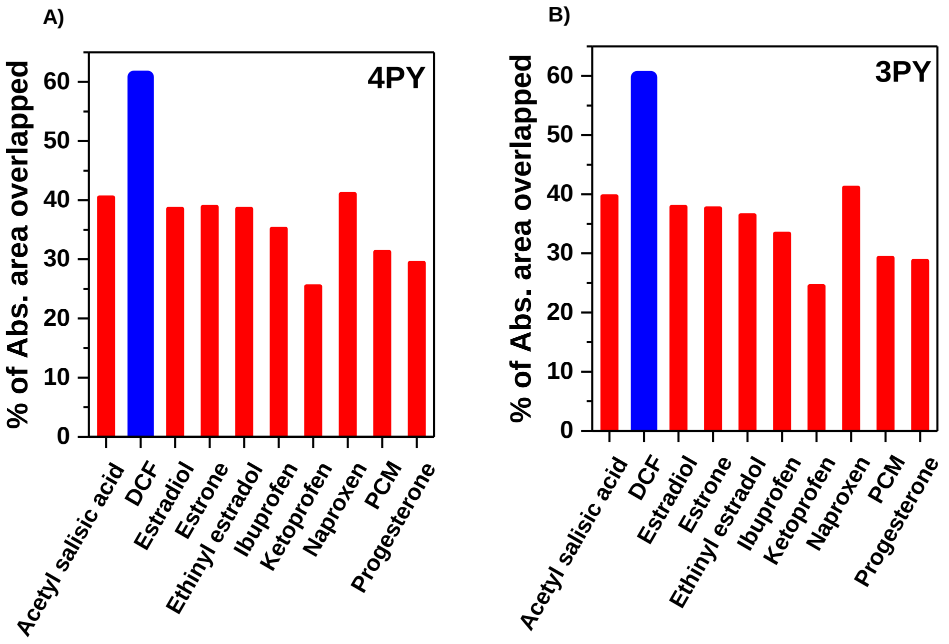

Figure S16: *The value represented here is the area of overlap region with area of excitation spectra band of 3PY (Right) and 4PY (Left) divided by area of individual absorbance band pharmaceutical compound.*

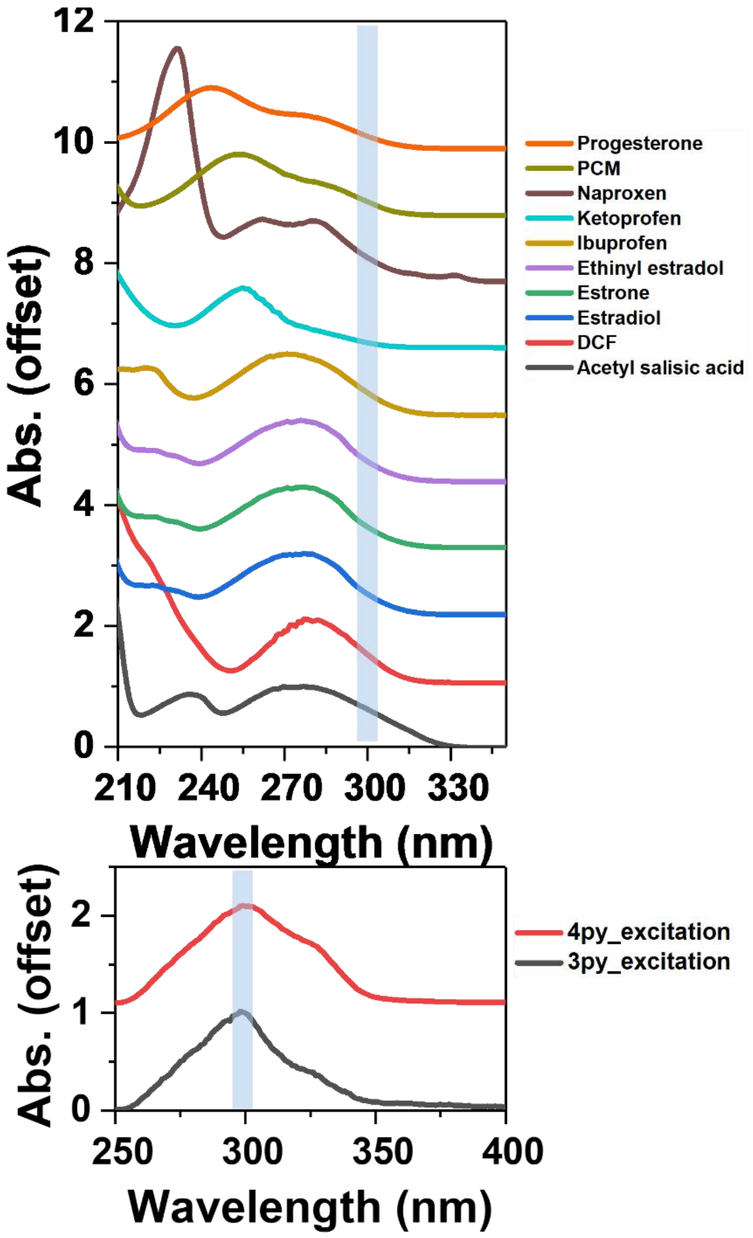

Figure S17: *The overlap of pharmaceutical compounds absorbance spectra with the excitation spectra of 3PY and 4PY. The compounds here are DCF, Estradiol, estrone, Ethinyl estradol, Ibuprofen, Ketoprofen, Naproxen, Progesterone and Acetyl salisic acid.*

Section 7: Sensing in real samples

The graph below shows the fluorescence intensity at 400 nm for all samples. The calibration plot for 3PY was adjusted by multiplying the overall profile by a constant factor of 1.73 to achieve a more uniform recovery ratio. In contrast, the 4PY case is presented without any adjustments. The constant factor for 3PY could be calculated by comparing the tangent drawn on calibration plot and observed plot. In general, we found out that the constant factor multiplied with 3PY is in the range of 1.7 to 2.4.

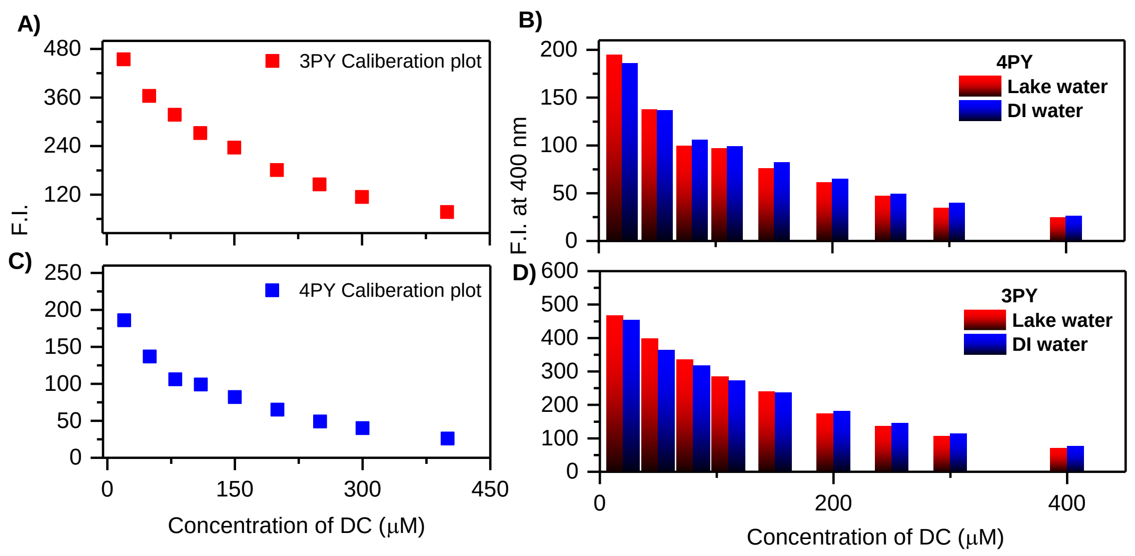

Figure S18: *A and C are the calibration plot for 3PY and 4PY respectively. The calibration plot for 3PY is multiplied by a constant 1.73. B and D is the fluorescence intensity profile monitored at 400 nm in the lake and deionized water.*

Section 8: Electrochemical studies

Cyclic voltammogram was employed to study the electrochemical behavior of 3PY/4PY/DC and to estimate its HOMO/LUMO energy levels [12]. The classic formula which states the relationship between HOMO energy level and onset potential of the material is shown as the following:

$$E_{HOMO} = -[E_{ox.(onset)} - E_{ox.(ferrocene)}] - 4.8 \text{ eV} \qquad \text{----------------------------}(13)$$

In the equation, 4.8 is the ferrocene reference energy level and $E_{ox.(ferrocene)}$ is the potential when ferrocene starts being oxidized (0.40 eV vs. Ag/AgCl). In order to calculate the LUMO energy level, the following two equations are further used:

$$E_{LUMO} = E_{HOMO} + E_{gap} \qquad \text{----------------------------}(14)$$

$$E_{gap} = hc/\lambda_{edge} \text{ ----------------------------}(15)$$

where E_{gap} is the optical bandgap of the compound, h is the Planck constant (6.63 ×10-34 m^2 kg s^{-1}), c is the speed of light (3×10^8 m s^{-1}) and λ_{edge} is the characteristic edge absorption. For the mechanistic analysis, we used the electrochemically derived HOMO and LUMO values shown in 4th and 5th column of the table.

Table S4: *Electrochemical parameter of 3PY/4PY/DCF.*

Compounds	E_{onset} (Ox) (V)	E_{onset} (Red) (V)	E_{HOMO} (eV)	E_{LUMO} (eV)	E_{LUMO}^{opt} (eV)	E_{gap} (CV) (eV)	E_{gap} (opt) (eV)
3PY	+1.44	-0.66	-5.84	-3.74	-2.39	2.10	3.45
4PY	+1.90	-0.72	-6.30	-3.68	-2.90	2.62	3.40
DCF	+0.48	-0.66	-4.88	-3.74	-1.01	1.14	3.87

- E_{onset} (Ox) and E_{onset} (Red) are the first potentials of the first oxidation and reduction processes.
- E_{HOMO} is calculated using Eq. 13
- E_{gap} (CV) is calculated as $E_{gap} = E_{LUMO} - E_{HOMO}$.
- CV = Cyclic Voltammetry.
- E_{HOMO}(Fc) = -4.8 eV; $E_{1/2\,(Ox)}$(Fc) = 0.40 V.
- E_{LUMO}^{opt} (eV) calculated by using Eq. 14.
- E_{lumo} is calculated as $E_{lumo} = -4.8 + (E_{1/2}^{(Ox)}(Fc) - E_{onset}\,(Red))$.

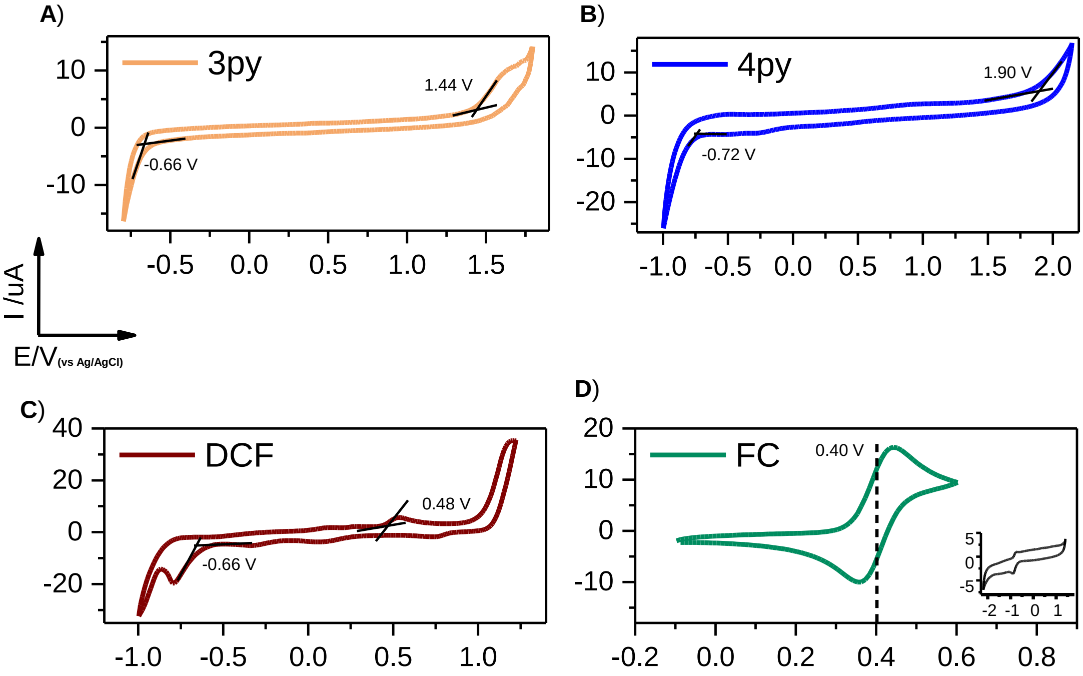

Figure S19: *A, B, C and D plot are the electrochemical response of 3PY, 4PY, DCF and Ferrocene respectively. The x axis is in Potential (Volt) with reference of Ag/AgCl and y axis is Current in micro ampere. The scanning was done with 100mV step size. The potential window was selected according to the compound.*

Section 9: Effect of alkali, alkaline earth, and heavy metal ions with 3PY and 4PY.

The potential interference of common metal cations by testing the fluorescence response of Probes **3PY** and **4PY** with various metal ions (500 µM). As shown in Figure S20, alkali and alkaline earth metals (Na^+, K^+, Ca^{2+}, Mg^{2+}) caused minimal fluorescence changes in both probes. However, heavy metal analytes like Fe^{2+}, Cu^{2+}significantly quenched the fluorescence of both probes, while Cadmium, Nickel, mercury and Cobalt showed no quenching for Probes **3PY** and **4PY**. While some metal ions interact with the probes, their interference with DCF detection in typical water samples may be limited due to potentially lower environmental concentrations.

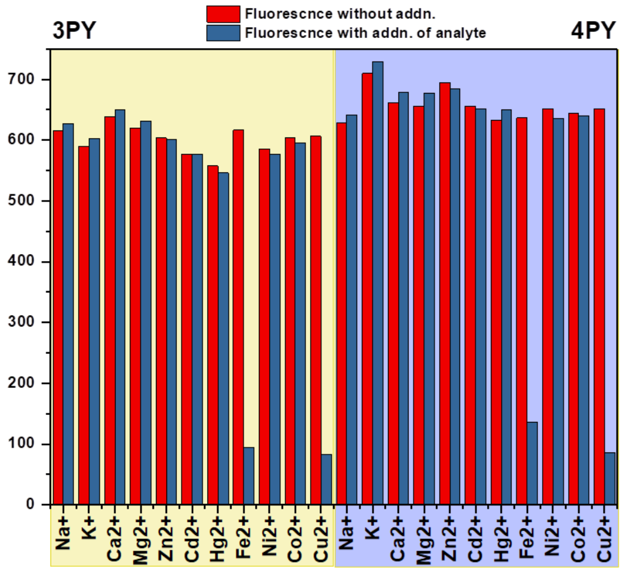

Figure S20: *Effect of alkali, alkaline earth, and heavy metal ions with 3PY and 4PY. (Concentration of probe and cations is 5 µM and 500 µM respectively.)*

Section 10: Fluorescent spectra of DCF and standard deviation of the measured fluorescence intensities at concentrations close to the calculated LOD.

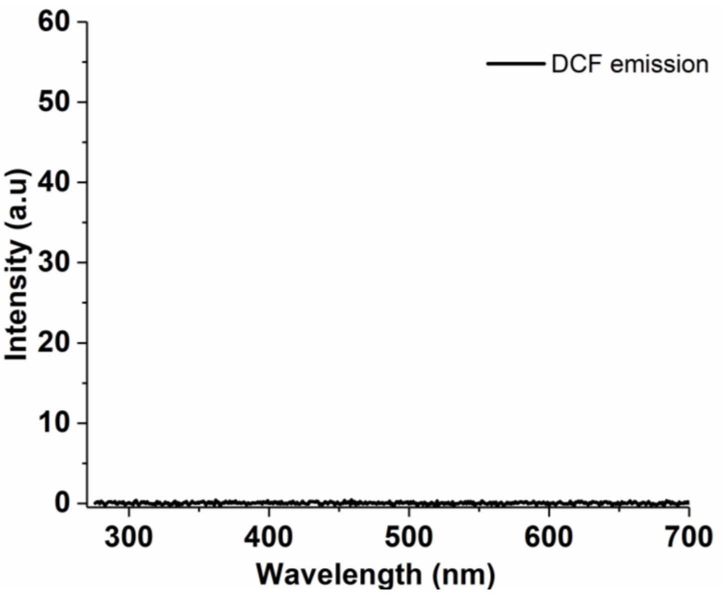

Figure S21: *Emission spectra of DCF (2 mM) in ethanol (λ_{ex}=280 nm).*

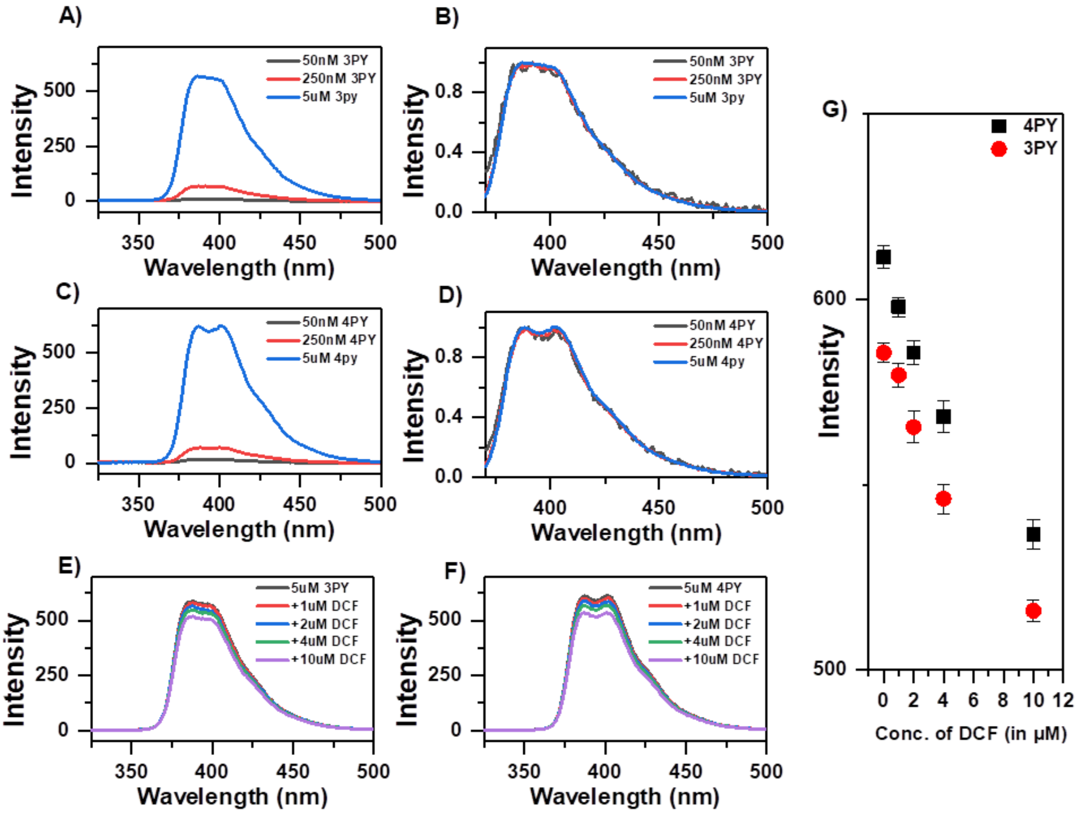

Figure S22: A) Fluorescent spectra of 3PY at different concentrations starting from 50nM to 5 μM in DMSO at (λ_{ex}= 300nm). B) Normalized fluorescent spectra of 3PY (Fig.A). C) Fluorescent spectra of 4PY at different concentrations starting from 50nM to 5 μM in DMSO at (λ_{ex}= 300nm). D) Normalized fluorescent spectra of 4PY (Fig.C). E) Fluorescent spectra of 3PY (5 μM) with different concentrations of DCF starting from 1 μM to 10 μM in DMSO at (λ_{ex}= 300nm). F) Fluorescent spectra of 4PY (5 μM) with different concentrations of DCF starting from 1 μM to 10 μM in DMSO at (λ_{ex}= 300nm). G) Standard deviation of the measured fluorescence intensities at DCF concentrations close to the calculated LOD (1 μM to 10 μM) in DMSO with 5 μM of probe excited at 300nm (seven measurements).

References

1. Würth, C., Grabolle, M., Pauli, J., Spieles, M., and Resch-Genger, U. (2013) Relative and absolute determination of fluorescence quantum yields of transparent samples. *Nat Protoc*, **8** (8), 1535–1550.

2. Puchalski, M.M., M.M.J.& von W.R. (1991) Assessment of inner filter effect corrections in fluorimetry. *Fresenius J Anal Chem*, **340**, 341–344.

3. Fonin, A. V., Sulatskaya, A.I., Kuznetsova, I.M., and Turoverov, K.K. (2014) Fluorescence of dyes in solutions with high absorbance. Inner filter effect correction. *PLoS One*, **9** (7).

4. van Slageren, R., den Boef, G., and van der Linden, W.E. (1973) The signal vs. concentration relationships in fluorimetry. *Talanta*, **20** (5), 501–512.

5. C. A. Parker, W.T.R. (1962) Fluorescence spectrometry. A review. *Analyst*, **87**, 83–111.

6. Kumar Panigrahi, S., and Kumar Mishra, A. (2019) Inner filter effect in fluorescence spectroscopy: As a problem and as a solution. *Journal of Photochemistry and Photobiology C: Photochemistry Reviews*, **41**, 100318.

7. C. A. Parker, W.J.B. (1957) Some experiments with spectrofluorimeters and filter fluorimeters. *Analyst*, **82**, 606–618.

8. Mikael Kubista, R.S.S.E. and B.A. (1994) Experimental correction for the inner-filter effect in fluorescence spectra. *Analyst*, **119**, 417–419.

9. Joseph R. Lakowicz (2006) *Principles of Fluorescence Spectroscopy*, Springer New York, NY.

10. Lakowicz, J.R. (2006) *Instrumentation for Fluorescence Spectroscopy*, Springer, Boston, MA.

11. Mäntele, W., and Deniz, E. (2017) UV–VIS absorption spectroscopy: Lambert-Beer reloaded. *Spectrochim Acta A Mol Biomol Spectrosc*, **173**, 965–968.

12. Sworakowski, J. (2018) How accurate are energies of HOMO and LUMO levels in small-molecule organic semiconductors determined from cyclic voltammetry or optical spectroscopy? *Synth Met*, **235**, 125–130.
